# Influence of the MUC1 Cell Surface Mucin on Gastric Mucosal Gene Expression Profiles in Response to *Helicobacter pylori* Infection in Mice

**DOI:** 10.3389/fcimb.2020.00343

**Published:** 2020-07-24

**Authors:** Yong H. Sheng, Garrett Z. Ng, Kim M. Summers, Alison L. Every, Gareth Price, Sumaira Z. Hasnain, Philip Sutton, Michael A. McGuckin

**Affiliations:** ^1^Inflammatory Disease Biology and Therapeutics Group, Mater Research Institute – The University of Queensland, Translational Research Institute, Woolloongabba, QLD, Australia; ^2^Centre for Animal Biotechnology, Faculty of Veterinary and Agricultural Sciences, The University of Melbourne, Parkville, VIC, Australia; ^3^Genetics, Genomics & Transcriptomics of Disease Group, Mater Research Institute – The University of Queensland, Translational Research Institute, Woolloongabba, QLD, Australia; ^4^QCIF Facility for Advanced Bioinformatics, Institute of Molecular Bioscience, The University of Queensland, St Lucia, QLD, Australia; ^5^Mucosal Immunology, Murdoch Children's Research Institute, Royal Children's Hospital, Parkville, VIC, Australia; ^6^Department of Paediatrics, Faculty of Medicine Dentistry and Health Sciences, The University of Melbourne, Parkville, VIC, Australia; ^7^Faculty of Medicine Dentistry and Health Sciences, The University of Melbourne, Parkville, VIC, Australia

**Keywords:** MUC1, *Helicobacter pylori*, infection, gene expression, lipid metabolism, SPINK1

## Abstract

The cell surface mucin MUC1 is an important host factor limiting *Helicobacter pylori (H. pylori*) pathogenesis in both humans and mice by providing a protective barrier and modulating mucosal epithelial and leukocyte responses. The aim of this study was to establish the time-course of molecular events in MUC1-modulated gene expression profiles in response to *H. pylori* infection in wild type (WT) and MUC1-deficient mice using microarray-determined mRNA expression, gene network analysis and Ingenuity Pathway Analysis (IPA). A time-course over the first 72 h of infection showed significantly higher mucosal loads of bacteria at 8 h of infection in *Muc1*^−/−^ mice compared with WT, confirming its importance in the early stages of infection (*P* = 0.0003). Microarray analysis revealed 266 differentially expressed genes at one or more time-points over 72 h in the gastric mucosa of *Muc1*^−/−^ mice compared with WT control using a threshold of 2-fold change. The SPINK1 pancreatic cancer canonical pathway was strongly inhibited in *Muc1*^−/−^ mice compared with WT at sham and 8 h infection (*P* = 6.08E-14 and *P* = 2.25 E-19, respectively) but potently activated at 24 and 72 h post-infection (*P* = 1.38E-22 and *P* = 5.87E-13, respectively). The changes in this pathway are reflective of higher expression of genes mediating digestion and absorption of lipids, carbohydrates, and proteins at sham and 8 h infection in the absence of MUC1, but that this transcriptional signature is highly down regulated as infection progresses in the absence of MUC1. Uninfected *Muc1*^−/−^ gastric tissue was highly enriched for expression of factors involved in lipid metabolism and 8 h infection further activated this network compared with WT. As infection progressed, a network of antimicrobial and anti-inflammatory response genes was more highly activated in *Muc1*^−/−^ than WT mice. Key target genes identified by time-course microarrays were independently validated using RT-qPCR. These results highlight the dynamic interplay between the host and *H. pylori*, and the role of MUC1 in host defense, and provide a general picture of changes in cellular gene expression modulated by MUC1 in a time-dependent manner in response to *H. pylori* infection.

## Introduction

Chronic infection with the gram-negative bacterium *Helicobacter pylori (H. pylori)* induces a chronic gastritis in susceptible individuals, which can lead to gastric ulcers, and adenocarcinoma, a malignancy of the glandular epithelium of the stomach. Despite declining prevalence of infection in the Western world, gastric cancer remains one of the most common and deadly cancers worldwide, and is the 5th most common neoplasm and the 3rd most deadly cancer, with an estimated 783,000 deaths in 2018 (Rawla and Barsouk, 2019). Only a fraction of individuals infected with *H. pylori* will develop these associated pathologies, and this variability is attributed to a mixture of environmental, bacterial, and host factors.

One host factor linked with susceptibility to *Helicobacter*-associated gastritis and gastric cancer is allelic variation in the gene that encodes the MUC1 mucin. MUC1 cell surface-mucin is densely present on the apical membrane of most mucosal epithelial cells, including the gastric mucosa where it is very highly expressed. MUC1 is a dominant constituent of the glycocalyx (McGuckin et al., 2011; Sheng et al., 2012), with the full-length form of MUC1 consisting of two non-covalently-bound subunits: a long N-terminal extracellular domain and a short C-terminal cytoplasmic domain. MUC1 is also expressed by some leukocytes, including monocyte/macrophages, dendritic cells, and activated T cells (Wykes et al., 2002). Several epidemiologic studies have linked *MUC1* polymorphisms in humans with susceptibility to *H. pylori*-induced disease (Carvalho et al., 1997; Vinall et al., 2002), suggesting a direct effect of *MUC1* polymorphisms on the development of *Helicobacter*-associated pathology. We have shown that mice deficient in MUC1 are more susceptible to infection by *H. pylori* and that differences emerge very early in infection and are sustained, with characteristically more severe chronic inflammation (McGuckin et al., 2007). Mechanistically, we have shown that MUC1 in gastric epithelial cells limits *H. pylori* infection both by steric hindrance and by acting as a releasable decoy (Linden et al., 2009), and that MUC1 in macrophages suppresses inflammation by negatively regulating the inflammasome (Ng et al., 2016). However, the molecular network pathway by which this mucin limits bacterial pathogenesis has not been fully elucidated, and differences in the very early stages of infection have not been explored. Therefore, the aim of this study was to elucidate the time-course of molecular events and dynamic networks in gastric tissue from *Muc1*^−/−^ and wild type (WT) mice in response to the very early stages of *H. pylori* infection, and to validate key target genes involved in the molecular changes in gastric tissue during *H. pylori* infection.

## Materials and Methods

### *H. pylori* Culture

*H. pylori* strain SS1 (Lee et al., 1997), was initially cultured on horse blood agar plates [Blood Agar Base No. 2, 0.02% Amphostat, and Skirrow's Selective Supplements [Oxoid, Basingstoke, United Kingdom] and 5% horse blood [Biolab, Clayton, Australia]] in an anaerobic jar with a microaerophilic gas generating kit (Oxoid) for 2 days at 37°C. Prior to infection of mice, *H. pylori* were cultured in brain heart infusion broth (BHI; Oxoid) containing 5% horse serum (Sigma, Castle Hill, Australia), 0.02% Amphostat, and Skirrow's Selective Supplements under microaerophilic conditions for 24 h at 37°C.

### Murine Infection Experiments

*Muc1*^−/−^ [derived and kindly provided by Sandra Gendler, Mayo Clinic Spicer et al., 1995] and wild-type (WT) mice, all on a 129/SvJ background, were bred within the Veterinary Science animal house, University of Melbourne, and genotyped as described (Spicer et al., 1995). All experiments involved age-matched female mice and were performed under Animal Ethics Committee approval (University of Melbourne; AEEC No. 06205). Mice were infected intragastrically once with 10^7^ *H. pylori* SS1 suspended in 0.1 mL BHI and groups (*n* = 3 of each genotype) were euthanased after 8, 24, and 72 h for collection of gastric tissues to determine bacterial abundance and gastric gene expression. Control uninfected (sham) mice were mock-infected with 0.1 mL sterile BHI and sampled after 8, 24, and 72 h mock-infection (*n* = 3 of each genotype).

### Determination of Infection Levels

*H. pylori* infection levels within mouse gastric tissues were quantified by colony-forming assay. Briefly, stomachs were opened along the inner curvature and bisected longitudinally. One half was placed in BHI and homogenized (GmbH Polytron homogeniser, Kinematica, Switzerland) and the other half rapidly frozen for RNA extraction. Ten-fold serial dilutions were prepared in BHI broth and aliquots spread over GSSA selective agar plates [Blood Agar Base No. 2 with 5% horse blood, vancomycin (12 mg/mL), polymyxin B (0.40 mg/mL), bacitracin (24 mg/mL), nalidixic acid (1.3 mg/mL), and amphotericin B (3.75 mg/mL), all from Sigma]. After 5 days culture as above, colonies were counted, and the number of colony-forming units was calculated per stomach (McGuckin et al., 2007). Colonies were confirmed to be *H. pylori* by the rapid urease test as previously described (Sutton et al., 2000).

### RNA Preparation and Quality Control

RNA was extracted from each longitudinally bisected half stomach of each mouse using Trizol (Invitrogen) and further purified on RNeasy columns (Qiagen). RNA integrity (RNA integrity number >8) was verified using a Bio-analyzer (Agilent) and RNA was stored at −70°C prior to further analysis by microarray and reverse-transcription-quantitative PCR (RT-qPCR).

### Time-Course Microarray Assays

Equal quantities of RNA from mice of the same genotype and time point were pooled for microarray assays. For sham-infected mice, equal quantities of RNA from mice of the same genotype and all three time points were pooled as control uninfected mice. Samples were labeled for GeneChip analysis using the One-Cycle Target Labeling and Control Reagents (Affymetrix). The gene expression array used was the Affymetrix Mouse Gene 1.0 ST array. All steps of target labeling, hybridization, and scanning were performed according to the manufacturer's protocol. The entire microarray dataset is available in Supplementary Table 1.

### Molecular Network Analysis

We used several gene ontology-based databases-to examine expression of genes across the time course of response to infection as follows;

#### Ingenuity Knowledge Base Network Analysis

Ingenuity Knowledge Base network analysis was conducted using Ingenuity Pathway Analysis (IPA) which is based on the QIAGEN Knowledge Base (QIAGEN Inc., https://digitalinsights.qiagen.com/products-overview/discovery-insights-portfolio/analysis-and-visualization/qiagen-ipa/), a large repository of biological interactions between proteins, RNAs, genes, isoforms, metabolites, complexes, cells, tissues, drugs and diseases, manually curated by experts based on over 3.58 million published studies. The Knowledge Base includes biological interaction data on 19,600 human and 14,700 mouse genes. For each time-point, IPA was used to construct molecular networks of direct physical, transcriptional and enzymatic interactions. Genes identified as differentially expressed were overlaid onto the interactome. Focus genes, which had direct interactions with other genes in IPA, were identified. For each focus gene, the specificity of connections was calculated by the percentage of its connections to other significant genes. Each network was constrained to a maximum of 35 genes. Network scores were calculated based on statistical likelihood (Calvano et al., 2005). The score indicates the likelihood that the assembly of a set of focus genes in a network could not be explained by random chance alone. For each time-point, networks with a statistical likelihood score above 30 are presented.

#### Graphia Pro 1.4 Network Analysis

The network analysis tool Graphia Pro 1.4 (formerly BioLayout *Express*^3D^; http://biolayout.org.) (Theocharidis et al., 2009) was used to examine expression of genes across the time course of response to infection. Graphia Pro 1.4 clusters data based on similarity of gene expression pattern with nodes representing a data point and edges the relation between nodes. The results were filtered to remove genes with low dynamic range. 6016 genes where the maximum value across all samples was at least 1.5 times the minimum value across all samples were included in the final analysis. In a sample-to-sample analysis (similar to a principal components analysis) nodes represent samples and the network layout shows the similarity of samples based on the expression of all genes in the sample. A gene-to-gene analysis generates a gene coexpression network (GCN) (Wolfe et al., 2005) in which nodes represent genes and edges the correlation between them at or above the chosen threshold. The network layout shows the similarity of gene expression patterns across all samples. The Markov clustering algorithm (MCL) (van Dongen and Abreu-Goodger, 2012) identified groups of highly connected genes within the elements of the network. The inflation value was set at 1.7 to control the granularity of the clusters. GO term enrichment was assessed using Database for Annotation, Visualization and Integrated Discovery (DAVID v6.8; http://david.ncifcrf.gov) (Huang da et al., 2009a,b). Gene Annotation Tool to Help Explain Relationships (GATHER; https://changlab.uth.tmc.edu/gather/) (Chang and Nevins, 2006) and PANTHER (version of 3 January 2020; http://geneontology.org/) (Mi et al., 2019a,b). *Mus musculus* was used as the reference genome. GO terms were selected for biological processes (GOTERM_BP_DIRECT), cellular components (GOTERM_CC_DIRECT), and molecular function (GOTERM_MF_DIRECT).

### RT-qPCR Validation of Microarray

To validate microarray data, a selection of the most highly differentially expressed genes between *Muc1*^−/−^ mice and WT mice were measured independently by RT-qPCR using the same individual RNA samples. Total RNA (1 μg) from each individual sample was used for first strand cDNA synthesis using SuperScriptTM III reverse transcriptase (Life Technologies) following the manufacturer's instructions. Real-time PCR was performed on a Rotor-Gene 3000 cycler (Qiagen) by using SYBR ® Green I fluorescence (Life Technologies). The cycling conditions were: denaturation for 10 min at 95°C, followed by 40 amplification cycles of 20 s of denaturation at 94°C, 30 s of annealing at 60°C, and 30 s of extension at 72°C. To confirm the specificity of the amplified DNA, a melting curve was determined at the end of each run. The reaction efficiency was determined with a dilution series of cDNA containing the PCR products. Expression of the target genes was normalized to that of *Actb* (encoding β-actin) and the results presented as their ratios (arbitrary units). Control experiments were also performed to ensure that housekeeping gene expression was not differentially regulated under the experimental conditions employed.

The primers used for PCR were designed from Primer Bank (https://www.ncbi.nlm.nih.gov/tools/primer-blast/index.cgi) or using Oligoperfect Designer (Life Technologies), and their sequences to amplify the target genes are shown in Supplementary Table 2.

### Statistical Analysis

For microarray studies, statistical analysis was performed on the R platform using the limma package from Bioconductor. The False Discovery Rate (FDR) method and Fisher's Exact Test were used with a cut off for statistical significance of *P*-value of <0.05 and a fold expression change of 2. For the remainder of the study, statistical analyses were performed using Prism Software v5 (Graphpad) by using ordinary one-way ANOVA and *post-hoc* testing. No data were excluded from any analyses. The statistical test used and the sample sizes for individual analyses are provided within the figure legends.

## Results

### *H. pylori* Colonization Is Elevated Very Early in Infection in *Muc1*^-/-^ Mice

**Figure 1:**
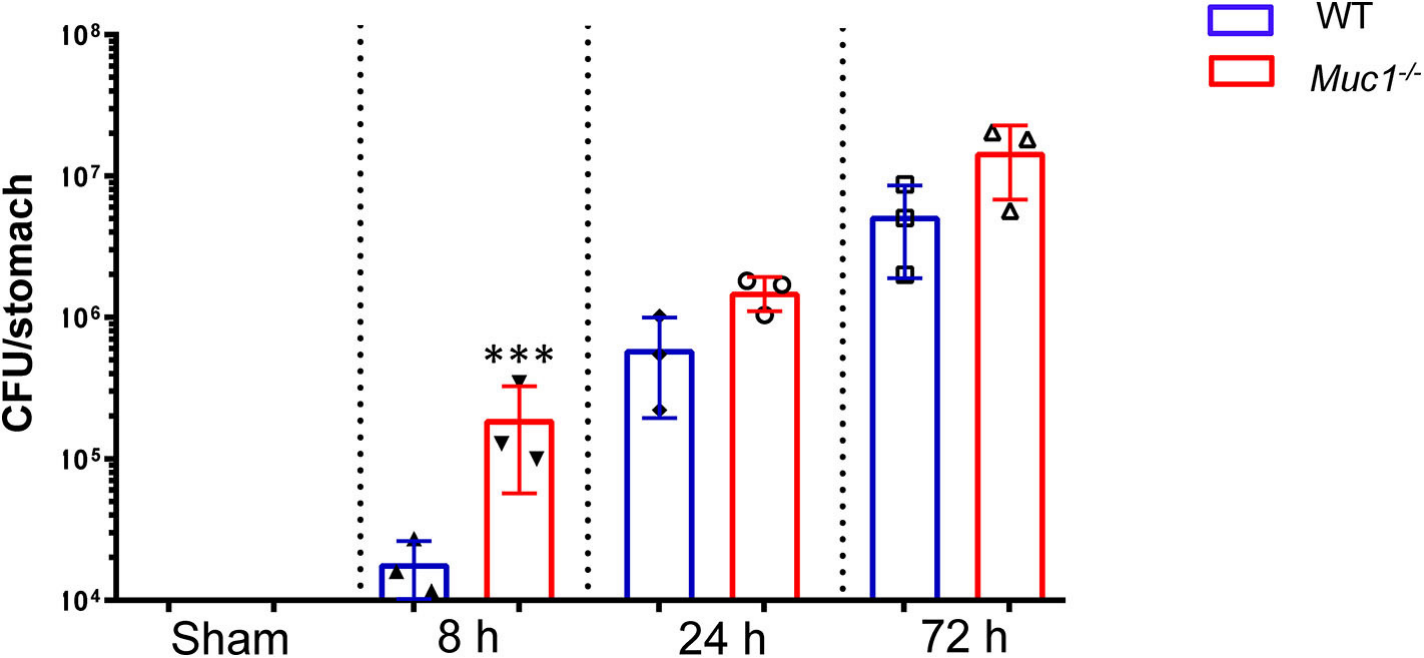
*H. pylori* colonization levels are elevated in *Muc1*^−/−^ mice compared with wild-type (WT) controls. WT or *Muc1*^−/−^ mice were infected with a single challenge of *H pylori*-SS1. At each time point, stomachs were removed, and bacterial colonization was determined by colony-forming assay. Statistics: *Muc1*^−/−^ vs. wild-type controls at each time point (Ordinary one-way ANOVA corrected for multiple comparisons by controlling the False Discovery Rate (FDR) using “Two-stage step-up method of Benjamini, Krieger, and Yekutieli;” *n* = 3, ***p < 0.001).

In wild type mice *H. pylori* colonization increased progressively over the first 3 days of infection (Figure 1). Consistent with our previous finding of higher *H. pylori* colonization in *Muc1*^−/−^ mice 24 h after infection, we found that *Muc1*^−/−^ mice displayed ~10-fold greater levels of *H. pylori* colonization in the stomach compared with wild-type (WT) mice as early as 8 h post-infection (*P* = 0.0003), consistent with a deficit in pre-existing or rapidly-induced innate defense. The higher CFU bacterial burden in the *Muc1*^−/−^ mice persisted, being 2.5- and 2.8-fold higher at 24 and 72 h post-infection (Figure 1).

### Identification and Categorization of Differentially Expressed Genes in the Gastric Tissue of *Muc1^−/−^* Mice

**Figure 2:**
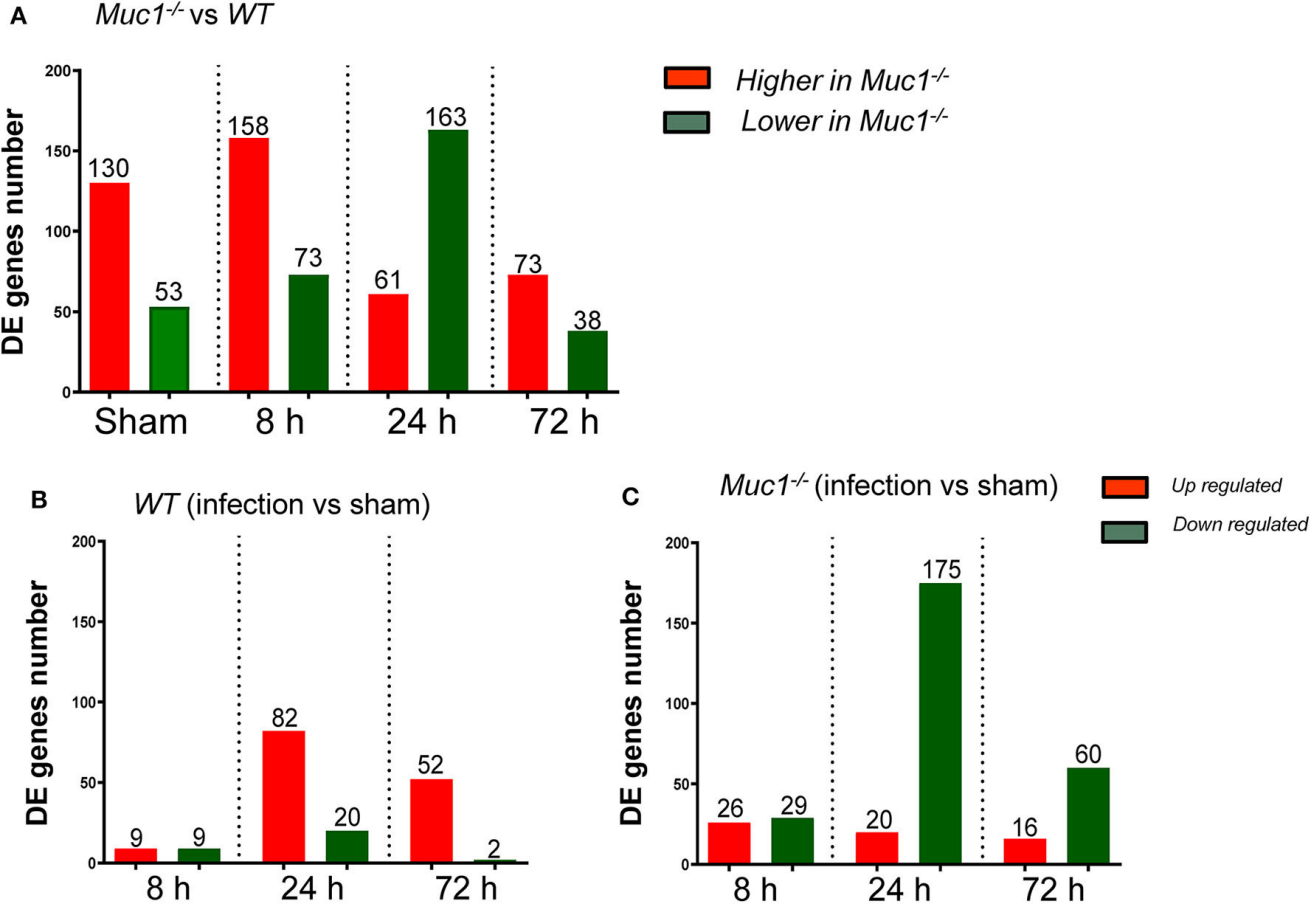
Differentially expressed (DE) genes in the gastric tissues across different time points of *H. pylori* infection. Control uninfected mice were sham-infected with 0.1 mL BHI and sampled after 8, 24, and 72 h mock-infection. Equal quantities of RNA from mice of the same genotype from the three time points were pooled as a single sham control for each of 8, 24, and 72 *H. pylori* infection. **(A)** DE genes between *Muc1*^−/−^ vs. wild type (WT) mice. **(B)** DE genes occurring in WT gastric tissues in response to *H. pylori* infection over the course of infection. **(C)** DE genes occurring in *Muc1*^−/−^ gastric tissues in response to *H*. pylori infection over the course of infection.

**Figure 3:**
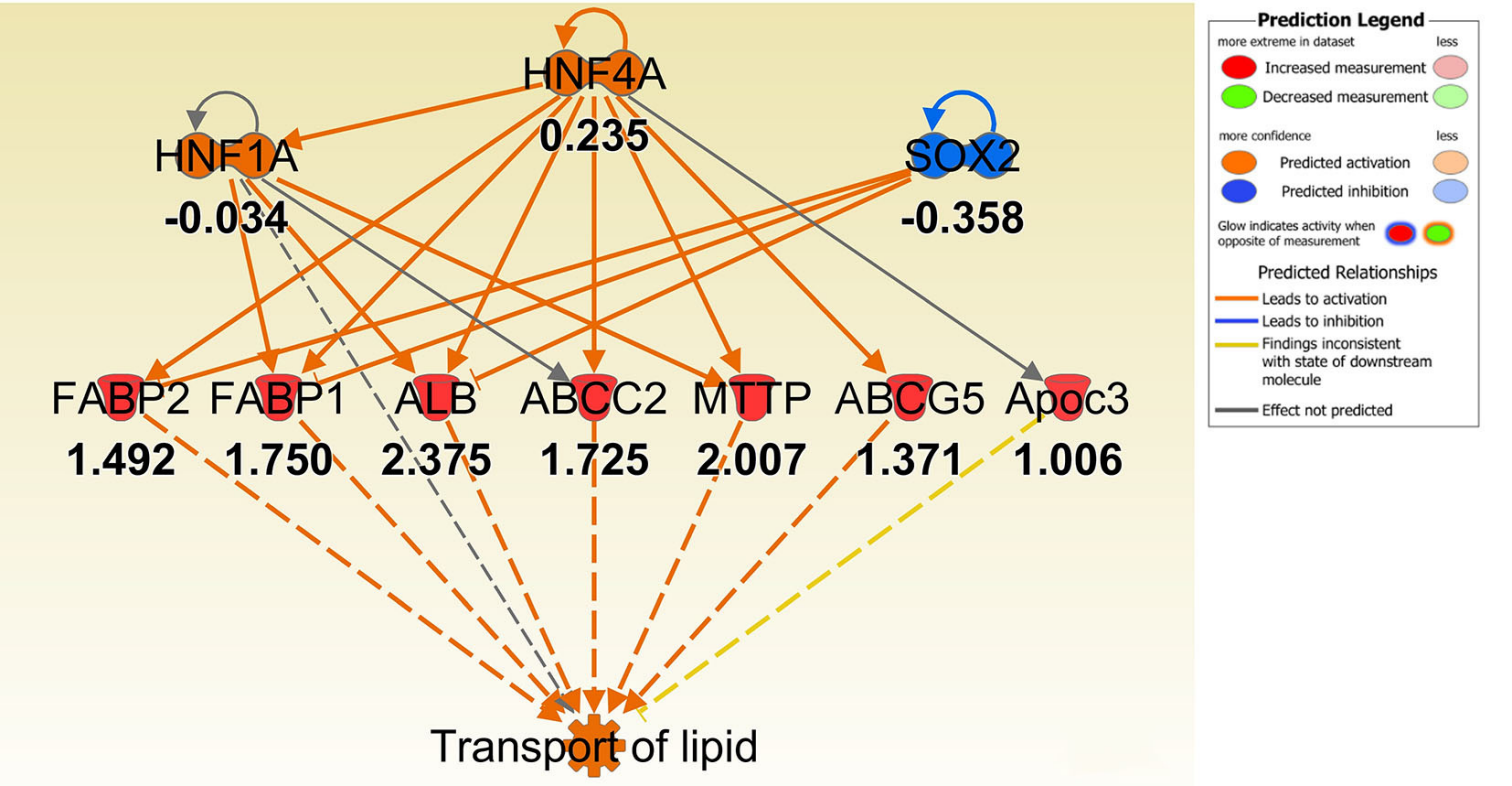
IPA regulator effect networks analysis of differentially expressed (DE) genes between Muc1^−/−^ vs. wild type (WT) gastric tissues before infection (The highest Consistency Score of regulator effect networks is shown). Upstream regulators are located at the top of the network, target genes are in the middle of network and predicated disease or function in the bottom of network. Log ratio fold changes of DE genes in our data set is showed underneath each gene (red color symbol indicates increased expression). The concept of Regulator Effects in IPA is that The Regulator Effects algorithm connects upstream regulators, dataset molecules and downstream functions or diseases affected in your dataset to generate a hypothesis that can explain how the activation or inhibition of an upstream regulator affects the downstream target molecule expression and the impact of the molecular expression on functions and diseases. The algorithm goes through one or more iterations to merge upstream and downstream results from the Upstream Regulator. The networks are merged only if the overlap of targets has possible statistical significance (Fisher's Exact Test p-value of <0.05). For each network, a Consistency Score is calculated that rewards for paths from regulator->target->disease or function that are consistent. Higher scoring hypotheses are those with more consistent causal paths represented by a high Consistency Score.

**Figure 4:**
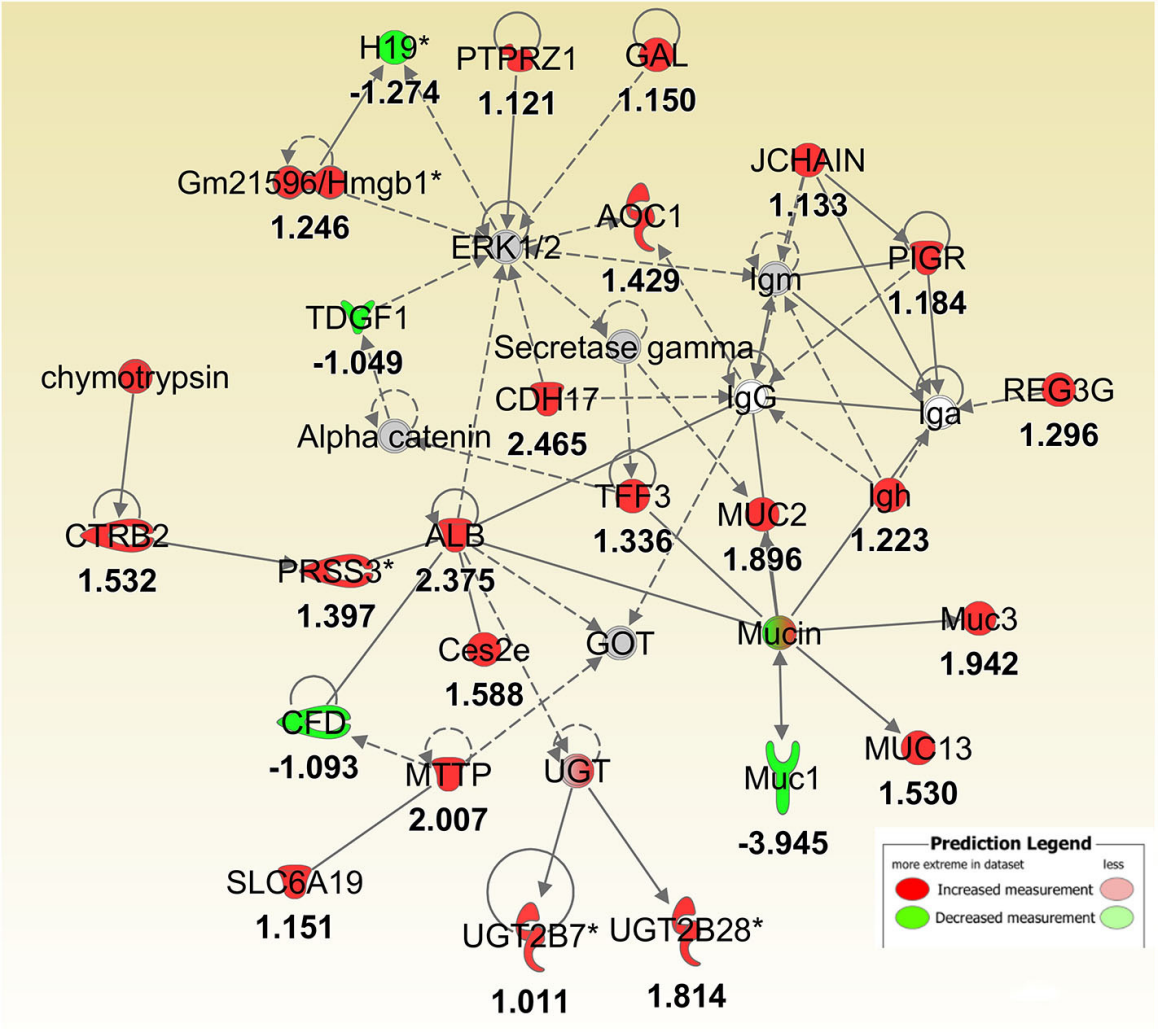
IPA molecular networks analysis of differentially expressed (DE) genes between *Muc1*^−/−^ vs. wild type gastric tissues before infection. Log ratio fold changes of DE genes in our data set is shown underneath each gene (- number or green symbol indicates decreased expression and otherwise/red indicates increased expression).

To better understand the molecular mechanism by which MUC1 mediates the inhibition of gastric *H. pylori* colonization, we applied a microarray approach to examine the effect of *Muc1* gene ablation on gastric gene expression in the absence of infection. We identified 183 transcripts that were differentially expressed (using a threshold of 2-fold change) in the gastric mucosa of uninfected *Muc1*^−/−^ mice (Figure 2A), and the top 10 differentially expressed genes are shown in Table 1. We further categorized the differentially expressed genes into specific functional groups according to gene ontology. Ingenuity Pathway Analysis (IPA) Entrez gene ontology annotation was used to determine the regulator effect network of MUC1-regulated genes (Figure 3) and the top regulators with consistence score 4.536 were *Hinf1a, Hinf4a*, and *Sox2* (Figure 3, Table 2). In line with the known functions of these upstream regulators, we found that their target genes in our data set were all increased in expression in *Muc1*^−/−^ gastric tissue, including *Fabp2, Fabp1*, ***Alb****, Abcc2*, ***Mttp****, Abcg5*, and *Apoc3* (Figure 3, genes with >4.5-fold changes are highlighted in bold). Increased expression of these genes is likely to lead to increased transport and metabolism of lipids (Figure 3, Table 2). Correspondingly, the top score (53) for network functions was attributed to digestive system development and function, humoral immune responses and organ development. This was followed by a score of 48 for network functions involved in lipid metabolism, molecular transport, and small molecule biochemistry (Table 3). These findings indicate that MUC1 modulates a lipid metabolic gene network, which is consistent with previous findings that MUC1 is a novel metabolic master regulator in human epithelial cancer cells (Mehla and Singh, 2014). In *Muc1* deficiency, other mucin genes including *Muc13, Muc2*, and *Muc3* were increased in expression in gastric tissues (Figure 4), although, given the other changes in gene expression and the differences in colonization, this was clearly unable to compensate functionally for loss of *Muc1*.

**Table 1:** Differentially expressed 10 top genes in the Gastric Muc1^−/−^ vs WT (KO/WT) or 72 h infection vs Sham (72h/0h) in WT or Muc1^−/−^ (KO).

**Sham (KO/WT)**			**8 h (KO/WT)**			**24 h (KO/WT)**			**72 h (KO/WT)**			**WT (72 h/0 h)**			**KO (72 h/0 h)**		
**Up molecules**	**Expr log ratio**	**Entrez gene name**	**Up molecules**	**Expr log ratio**	**Entrez gene** **name**	**Up molecules**	**Expr log ratio**	**Entrez gene name**	**Up molecules**	**Expr log ratio**	**Entrez gene** **name**	**Up molecules**	**Expr log ratio**	**Entrez gene name**	**Up molecules**	**Expr log ratio**	**Entrez gene name**
SI	3.165	Sucrase -isomaltase	CPB1	3.9	Carboxypeptidase B1	SLFN12L	2.69	Schlafen family member 12 like	ALB	3.67	Albumin	SI	3.65	Sucrase -isomaltase	HSPA1A/1B	1.75	Heat shock protein family A (Hsp70) member 1A
SLC13A1	2.658	Solute carrier family 13 member 1	REG1B	3.8	Regenerating family member 1 beta	Csprs	2.42	Component of Sp100-rs	Csprs	2.16	Component of Sp100-rs	SLC13A1	2.48	Solute carrier family 13 member 1	Csprs	1.55	Component of Sp100-rs
CYP3A11	2.584	Cytochrome P450, family 3, subfamily a, polypeptide 11	CPA2	3.8	Carboxypeptidase A2	IFI44	2.12	Interferon induced protein 44	IDO1	1.99	Indoleamine 2,3-dioxygenase 1	CPS1	2.47	Carbamoyl-phosphate synthetase 1	IDO1	1.47	Indoleamine 2,3-dioxygenase 1
SLC26A3	2.554	Solute carrier family 26 member 3	CPA1	3.7	Carboxypeptidase A1	IFIT1B	1.75	Interferon induced protein with tetratricopeptide repeats 1B	TRDN	1.9	Triadin	CDH17	2.43	Cadherin 17	Gm5431	1.28	
CPS1	2.553	Carbamoyl- phosphate synthetase 1	CTRL1	3.7	Chymotrypsin like 1	RSAD2	1.64	Radical S-adenosyl methionine domain containing 2	FGG	1.76	Fibrinogen gamma chain	MGAM	2.35	Maltase-glucoamylase	HBA1/HAA2	1.24	Hemoglobin subunit alpha 2
CDH17	2.465	Cadherin 17	ALB	3.5	Albumin	Trim30a/d	1.6	Tripartite motif-containing 30A	SERPINA1	1.72	Serpin family A member 1	SLC26A3	2.31	Solute carrier family 26 member 3	Trav8-2	1.2	T-cell receptor alpha variable 8-2
MGAM	2.4	Maltase- glucoamylase	CELA3B	3.5	Chymotrypsin like elastase 3B	USP18	1.58	Ubiquitin specific peptidase 18	IFIT3	1.65	Interferon induced protein with tetratricopeptide repeats 3	Igkv9-120	2.22	Immunoglobulin kappa chain variable 9-120	Olfr1274-ps	1.17	Olfactory receptor 1506
ALB	2.4	Albumin	PNLIP	3.4	Pancreatic lipase	Slfn2	1.55	Schlafen 2	Svs3a/Svs3b	1.6	Seminal vesicle secretory protein 3A	ALPP	2.198	Alkaline phosphatase, placental	Igkv4-55	1.16	Immunoglobulin kappa chain variable 4-55
CLCA4	2.3	Chloride channel accessory 4	2210010C04Rik	3.4	RIKEN cDNA 2210010C04 gene	LOC68395	1.55	Histocompatibility 2, Q region locus 6-like	Trim30a/d	1.59	Tripartite motif-containing 30A	CLCA4	2.14	Chloride channel accessory 4	FGG	1.15	Fibrinogen gamma chain
201010 6E10Rik	2.2		CTRB2	3.4	chymotrypsinogen B2	DHX58	1.53	DExH-box helicase 58	Gm5662	1.57		Muc3	2.12	Mucin 3, transmembrane	AHSG	1.13	Alpha 2-HS glycoprotein
**Down molecules**	**Expr log ratio**	**Entrez gene name**	**Down molecules**	**Expr log ratio**	**Entrez gene name**	**Down molecules**	**Expr log ratio**	**Entrez gene name**	**Down molecules**	**Expr log ratio**	**Entrez gene name**	**Down molecules**	**Expr log ratio**	**Entrez gene name**	**Down molecules**	**Expr log ratio**	**Entrez gene name**
Muc1	3.9	Mucin 1, transmembrane	Muc1	3.96	Mucin 1, transmembrane	CPA1	3.94	Carboxypeptidase A1	CTRB2	4.27	Chymotrypsinogen B2	Olfr960	1.39	Olfactory receptor 960	PNLIP	5.68	Pancreatic lipase
Defb4	1.7	Defensin beta 4	UXT	1.61	Ubiquitously expressed prefoldin like chaperone	CPA2	3.898	Carboxypeptidase A2	CPB1	3.76	Carboxypeptidase B1	DGLUCY	1.36	D-glutamate cyclase	CPB1	5.45	Carboxypeptidase B1
Olfr591	1.6	Olfactory receptor 591	Gm10573	1.52	Palmitoyl-protein thioesterase 1 pseudogene	CPB1	3.856	Carboxypeptidase B1	PNLIP	3.76	Pancreatic lipase	Olfr591	1.36	Olfactory receptor591	CTRB2	5.45	Chymotrypsinogen B2
Acnat	1.6	Acyl-coenzyme A amino acid N-acyltransferase 2	27000 79J08Rik	1.25		PNLIP	3.8	Pancreatic lipase	Muc1	3.52	Mucin 1, transmembrane	Acnat1/2	1.33	Acyl-coenzyme A amino acid N-acyltransferase 2	CPA1	5.16	Carboxypeptidase A1
1700010D01 Rik/Gm5941	1.5		impdh2	1.14	Inosine monophosphate dehydrogenase 2	221001 0C04Rik	3.5		CPA1	3.48	Carboxypeptidase A1	OR5K1	1.31	Olfactory receptor family 5 subfamily K member 1	221001 0C04Rik	5.05	
GJD2	1.5	Gap junction protein delta 2	Elobl	1.13	Elongin B-like	CTRL	3.49	Chymotrypsin like 1	2210010C04Rik	3.35		Olfr469	1.31	Olfactory receptor 469	CTRL	4.5	Chymotrypsin like 1
Cym	1.5	Chymosin	Eppin	1.07	Epididymal peptidase inhibitor	Muc1	3.48	Mucin 1, transmembrane	CTRL	3.22	Chymotrypsin like 1	Olfr517	1.28	Olfactory receptor 517	CPA2	4.41	Carboxypeptidase A2
TRDN	1.4	Triadin	ZNF14	1.05	Zinc finger protein 14	CELA3b	3.47	Chymotrypsin like elastase 3B	CPA2	2.796	Carboxypeptidase A2	MRGPRX3	1.22	MAS related GPR family member X3	CEL	3.64	Carboxyl ester lipase
Zfp947/Zfp995	1.3	Zinc finger protein 995	SRGAP2	1.05	SLIT-ROBO Rho GTPase activating protein 2	CTRB2	3.44	Chymotrypsinogen B2	CEL	2.2	Carboxyl ester lipase	IDO1	1.21	Indoleamine 2,3-dioxygenase 1	CELA3b	3.43	Chymotrypsin like elastase 3B
Olfr1274	1.3	Olfactory receptor 1274	Psg18	1.01	Pregnancy specific glycoprotein 18	CEL	3.21	Carboxyl ester lipase	CELA3b	1.88	Chymotrypsin like elastase 3B	Klra7	1.2	Killer cell lectin-like receptor, subfamily A, member 4	PRSS2	3.01	Serine protease 2

*Citrate transport; ion transport; sodium ion transport; succinate transmembrane transport; sulfate transport; transmembrane transport. Number in green indicates decreased expression and red indicates increased expression*.

**Table 2:** Gastric Muc1^−/−^ vs. WT (KO/WT) regulator effects^*^ at different time points.

**Time**	**Consistency score**	**Node total**	**Regulator total**	**Regulators**	**Target total**	**Target molecules in dataset**	**Diseases and functions**
Sham (KO/WT)	4.536	11	3	HNF1A, HNF4A, SOX2	7	ABCC2, ABCG5, ALB, Apoc3, FABP1, FABP2, MTTP	Transport of lipid
8 h (KO/WT)	16.028	30	7	FOXA2, HNF1A, HNF4A, Ncoa-Nr1i2-Rxra, Ncoa-Nr1i3-Rxra, OSM, PXR ligand-PXR-Retinoic acid-RXRα	18	ABCC2, ALB, APOA4, APOC2, Apoc3, CXCL3, CYP2C8, CYP2C9, CYP2E1, CYP3A5, FABP1, FABP2, MTTP, MUC2, SCD, SLC5A1, SLPI, VDR	Abnormality of large intestine, conversion of lipid, enteritis, fatty acid metabolism, metabolism of vitamin
8 h (KO/WT)	15.718	40	8	FOXA2, miR-12201-5p (and other miRNAs w/seed GGAAGGA), miR-1293 (and other miRNAs w/seed GGGUGGU), Ncoa-Nr1i2-Rxra, Ncoa-Nr1i3-Rxra, NCOR-LXR-Oxysterol-RXR-9 cis RA, PPARGC1A, PXR ligand-PXR-Retinoic acid-RXRα	24	ABCC2, ADA, ALB, ALPI, APOA4, APOC2, Apoc3, CA3, CYP2C8, CYP2C9, CYP3A5, FABP1, GAL, GUCA2B, MME, MTTP, MUC2, PNLIPRP1, SCD, SERPINA1, SERPINB4, SLC2A2, SLC46A1, VDR	Disorder of blood pressure, fatty acid metabolism, inflammation of absolute anatomical region, inflammation of organ, metabolism of terpenoid, metabolism of vitamin, synthesis of lipid, transport of molecule
24 h (KO/WT)	165.333	75	63	ACKR2, BTK, CGAS, DDX58, DNASE2, DOCK8, FADD, FZD9, GAPDH, IFIH1, Ifn, IFN Beta, IFN type 1, IFNA1/IFNA13, IFNA2, IFNA4, Ifnar, IFNAR1, IFNB1, IFNE, IFNG, IFNL1, IFNL3, IFNL4, IL1B, IL1RN, IL21, IL4, Interferon alpha, IRF1, IRF3, IRF4, IRF5, IRF7, JAK, JAK1/2, MAPK1, MAVS, MYD88, NFATC2, NSD2, PAF1, PML, PNPT1, PRL, PTGER4, SAMSN1, SASH1, SOCS1, SP110, SPI1, STAT1, STAT2, STAT6, TGM2, TICAM1, TLR3, TLR4, TLR7, TLR9, TMEM173, TNF, TRIM24	9	CEL, Igtp, ISG15, Mx1, OAS1, OAS3, Oasl2, RSAD2, ZBP1	Relapsing-remitting multiple sclerosis, replication of viral replicon, replication of virus
24 h (KO/WT)	1.000	6	4	IFNB1	4	DHX58, Igtp, Irgm1, STAT1	Quantity of IL-12 in blood
72 h (KO/WT)	10.156	22	6	DNASE2, IKBKG, IL17A, SNCA, STAT2, TLR3	14	Defb3, DHX58, GBP2, IFIT3, IL33, IRF7, ISG15, Mx1, OAS2, OAS3, Oasl2, RSAD2, USP18, ZBP1	Infection of mammalia, viral infection
72 h (KO/WT)	4.333	14	4	IFNB1, IKBKG, IL17A, SNCA	9	Defb3, DHX58, GBP2, Igtp, IL33, IRF7, Irgm1, ISG15, USP18	Infection of mammalia

Regulator effects^*^: The concept of Regulator Effects in IPA is that The Regulator Effects algorithm connects upstream regulators, dataset molecules, and downstream functions or diseases affected in your dataset to generate a hypothesis that can explain how the activation or inhibition of an upstream regulator affects the downstream target molecule expression and the impact of the molecular expression on functions and diseases. The algorithm goes through one or more iterations to merge upstream and downstream results from the Upstream Regulator. The networks are merged only if the overlap of targets has possible statistical significance (Fisher's Exact Test p-value of <0.05). For each network, a Consistency Score is calculated that rewards for paths from regulator->target->disease or function that are consistent. Higher scoring hypotheses are those with more consistent causal paths represented by a high Consistency Score.

**Table 3:** Gastric *Muc1*^−/−^ vs. WT (KO/WT) molecule networks^*^ at different time points.

**Time**	**Molecules in network**	**Score**	**Focus molecules**	**Top diseases and functions**
Sham (KO/WT)	ALB, Alphacatenin, AOC1, CDH17, Ces2e, CFD, chymotrypsin, CTRB2, ERK1/2, GAL, Gm21596/Hmgb1, GOT, H19, Iga, IgG, Igh, Igm, JCHAIN, MTTP, Muc1, MUC13, MUC2, Muc3, Mucin, PIGR, PRSS3, PTPRZ1, REG3G, Secretase gamma, SLC6A19, TDGF1, TFF3, UGT, UGT2B28, UGT2B7	53	25	Digestive System Development and Function, Humoral Immune Response, Organ Development
Sham (KO/WT)	2210010C04Rik, ABCG5, Akt, ALDOB, Apoc3, carboxypeptidase, CPA1, CPA2, CPB1, CYP2E1, Cyp3a25 (includes others), CYP3A5, DBP, Defa3 (includes others), Defa6, FABP1, FABP2, Growth hormone, HDL, HDL-cholesterol, hemoglobin, HNF4α dimer, IL23, LDL-cholesterol, MEP1B, Nr1h, PRAP1, REG3A, SLC13A1, SLC2A2, SLC40A1, SLC5A1, TM4SF20, trypsin, Trypsinogen	48	23	Carbohydrate Metabolism, Lipid Metabolism, Small Molecule Biochemistry
Sham (KO/WT)	Ap1, ARG2, CCN3, CELA3B, CG, Collagen type I (complex), Creb, CTRC, CTRL, Defb4, elastase, ENPP3, GGT1, GJD2, Iglv1, IL1, KLK3, LDL, LYZ, MHC Class II (complex), MME, Mmp, Mug1/Mug2, NFkB (complex), P38 MAPK, Pro-inflammatory Cytokine, PRSS2, S100G, Serine Protease, SERPINA1, SERPINB10, SI, TAC1, TCF, Tnf (family)	39	20	Post-Translational Modification, Protein Degradation, Protein Synthesis
8 h (KO/WT)	1810009J06Rik/Gm2663, 2210010C04Rik, carboxypeptidase, chymotrypsin, Collagen type I (complex), CPA1, CPA2, CPB1, Crisp1/Crisp3, CTRB2, CTRC, CTRL, estrogen receptor, FABP1, Growth hormone, Iglv1, KLK3, MEP1B, NFkB (complex), PRAP1, Prss1 (includes others), PRSS2, PRSS3, S100G, Serine Protease, SERPINA1, SI, SLC7A9, TCF, TM4SF20, Tnf (family), trypsin, Trypsinogen, UXT, VDR	50	24	Post-Translational Modification, Protein Degradation, Protein Synthesis
8 h (KO/WT)	ADA, AHSG, Akt, ALB, CELA1, CELA3B, Ces2e, CPS1, Defa3 (includes others), Defa6, elastase, ENPP3, GOT, hemoglobin, Ige, IgG, Igm, IL12 (complex), LDL, MHC Class II (complex), Muc1, MUC13, MUC2, Muc3, Mucin, PIGR, SCD, Secretase gamma, Serpinb3b/Serpinb3c, SLC13A1, SLC40A1, SLC6A19, SLPI, Sos, TFF3	45	22	Cancer, Dermatological Diseases and Conditions, Organismal Injury and Abnormalities
8 h (KO/WT)	ABCC2, ALDOB, ALT, AOC1, APOA4, APOC2, Apoc3, ARG2, BHMT, CAR ligand-CAR-Retinoic acid-RXRα, CDH17, CUZD1, CYP2C8, CYP2C9, CYP2E1, CYP3A5, ERK1/2, GAL, HDL, HDL-cholesterol, Hnf3, HNF4α dimer, LDL-cholesterol, MAT1A, MTTP, Ncoa-Nr1i2-Rxra, Ncoa-Nr1i3-Rxra, NCOR-LXR-Oxysterol-RXR-9 cis RA, Nr1h, PXR ligand-PXR-Retinoic acid-RXRα, Rxr, triacylglycerol lipase, unspecific monooxygenase, VLDL, VLDL-cholesterol	32	17	Drug Metabolism, Lipid Metabolism, Small Molecule Biochemistry
24 h (KO/WT)	2210010C04Rik, 3830403N18Rik/Xlr, ABCC2, Akt, ALDOB, CAR ligand-CAR-Retinoic acid-RXRα, carboxypeptidase, chymotrypsin, CPA1, CPA2, CPB1, CTRB2, CTRC, CTRL, CYP2B6, Cyp3a25 (includes others), CYP3A5, FABP1, FABP2, HNF4α dimer, Igtp, Ncoa-Nr1i2-Rxra, Ncoa-Nr1i3-Rxra, P glycoprotein, PRSS1, Prss1 (includes others), PRSS2, PRSS3, PXR ligand-PXR-Retinoic acid-RXRα, Rxr, SLC13A1, TMPRSS15, trypsin, Trypsinogen, TTPA	47	23	Drug Metabolism, Endocrine System Development and Function, Lipid Metabolism
24 h (KO/WT)	ACE2, Alp, ALPI, ALPP, AMPK, Ap1, APOC2, ARG2, Collagen type I (complex), Collagen(s), ERK, G6PC, GBP4, Gm10768, Growth hormone, Immunoglobulin, Interferon alpha, LDL, MEP1B, Mup1 (includes others), Nr1h, Oasl2, PHF11, PRAP1, RNF213, RTP4, S100G, SI, SLFN12L, Slfn2, SPINK1, STAT1, Tgf beta, TM4SF20, UBE2F	47	23	Developmental Disorder, Gastrointestinal Disease, Hereditary Disorder
24 h (KO/WT)	2' 5' oas, AOC1, CCL25, CDH17, CUZD1, DHX58, ERK1/2, IDO1, IFI44, IFIT1B, IFIT3, Ifn, IFN alpha/beta, IFN Beta, IFN type 1, Ifnar, Interferon-α Induced, IRF, IRF7, Irgm1, Isg, ISG15, ISGF3, JAK1/2, Mx1, Mx2, Oas, OAS1, OAS2, OAS3, RSAD2, Sp100, Stat1-Stat2, TCF, ZBP1	39	20	Antimicrobial Response, Dermatological Diseases and Conditions, Inflammatory Response
72 h (KO/WT)	DHX58, ERK1/2, IDO1, Ifi202b, IFI44, IFIT1B, IFIT3, Ifn, IFN alpha/beta, IFN Beta, IFN type 1, Ifnar, Igtp, Interferon alpha, Interferon-α Induced, IRF, IRF7, Irgm1, Isg, ISG15, ISGF3, JAK, JAK1/2, Mx1, Mx2, NLRC5, OAS2, OAS3, Oasl2, RSAD2, RTP4, Sp100, TAP1, USP18, ZBP1	50	22	Antimicrobial Response, Connective Tissue Disorders, Inflammatory Response
72 h (KO/WT)	2210010C04Rik, AHSG, Akt, ALB, AMY2B, amylase, carboxypeptidase, CEL, CPA1, CPA2, CPB1, CTRB2, CTRL, Fibrinogen, GBP2, GBP3, HDL, KNG1, LDL, MHC Class II (complex), Mug1/Mug2, Nr1h, PNLIP, PNLIPRP1, Pro-inflammatory Cytokine, PRSS2, PRSS3, Pzp, Serine Protease, SERPINA1, SLFN12L, Tnf (family), triacylglycerol lipase, trypsin, Trypsinogen	47	21	Endocrine System Disorders, Gastrointestinal Disease, Immunological Disease
72 h (KO/WT)	2' 5' oas, Apol7e (includes others), Apol9a/Apol9b, APP, beta-estradiol, C2orf49, CELA3B, CFB, CPA2, CYP2C18, dehydroepiandrosterone sulfate, DGLUCY, DHX58, GBP4, Gm5662 (includes others), GP2, GRIN2A, GSDMC, IFNAR1, IL10RA, IRF1, Mcpt1, Muc1, Mx2, nitrogen, NR5A2, Oasl2, PPP2CA, RBPJL, RNASE1, Rnu5g, SYCN, Tlr11, Tlr12, TNF	36	17	Cellular Assembly and Organization, Molecular Transport, Small Molecule Biochemistry

Molecule Networks^*^: Molecule Networks in IPA is that Analysis Ready molecules which interact with other molecules in the Ingenuity Knowledge Base are considered “network eligible” and serve as “seeds” for generating networks. There may be some Analysis Ready molecules that will pass all the filtering criteria you set for your analysis but may not be network eligible because there is no known scientific evidence of these molecules directly or indirectly interacting with other molecules in the Ingenuity Knowledge base. The score is a numerical value used to rank networks according to their degree of relevance to the Network Eligible molecules in your dataset. The score takes into account the number of Network Eligible molecules in the network and its size, as well as the total number of Network Eligible molecules analyzed and the total number of molecules in the Ingenuity Knowledge Base that could potentially be included in networks. In the Networks view, networks are ordered according to their score, with the highest scoring network displayed at the top of the page.

### Differences in Activation of Molecular Networks Between WT and *Muc1^−/−^* Mice in Response to *H. pylori* Infection

Next a transcriptomic analysis was performed at 8, 24, and 72 h post-infection. For this purpose, we analyzed each time point individually, as well as in various combinations. A comparative analysis using a threshold of 2-fold difference identified a total of 174 differentially expressed (DE) genes in WT and 326 in *Muc1*^−/−^ mice in response to infection (Figures 2B,C). The number of DE genes was higher in *Muc1*^−/−^ mice than WT mice at 8 h post infection (Figures 2B,C); the higher number of DE genes could be a consequence of the higher colonization levels of *H. pylori* in *Muc1*^−/−^ mice. At 24 h infection, there were fewer up-regulated genes (20) in *Muc1*^−/−^ compared to WT gastric mucosa (80), despite the higher colonization levels of *H. pylori* (Figures 2B,C). A similar trend was observed for up-regulated genes at 72 h infection. In contrast, *Muc1*^−/−^ mice showed a greater number of down-regulated genes (173) than WT (20) after 24 h infection, and the number of down-regulated genes surpassed the number of up-regulated genes throughout the time course in *Muc1*^−/−^ mice (Figures 2B,C). A list of the top 10 DE genes is provided in Table 1. The number of DE genes at each time point in comparison to uninfected controls is illustrated in Figures 2B,C. The number of DE genes at each time point in the gastric tissue of *Muc1*^−/−^ compared with WT mice is illustrated in Figure 2A.

### Pathway and Network Functional Categories

In this section, rather than discussing genes individually, we have grouped differentially regulated genes into canonical pathways and associated network functions, identified the upstream factors likely to drive the differentially expressed genes in the network, and focused on the most highly statistically significant changes in the pathway and network.

### Temporal Changes in the SPINK 1 Pancreatic Cancer Pathway in WT and *Muc1^−/−^* Mice in Response to *H. pylori* Infection

**Figure 5:**
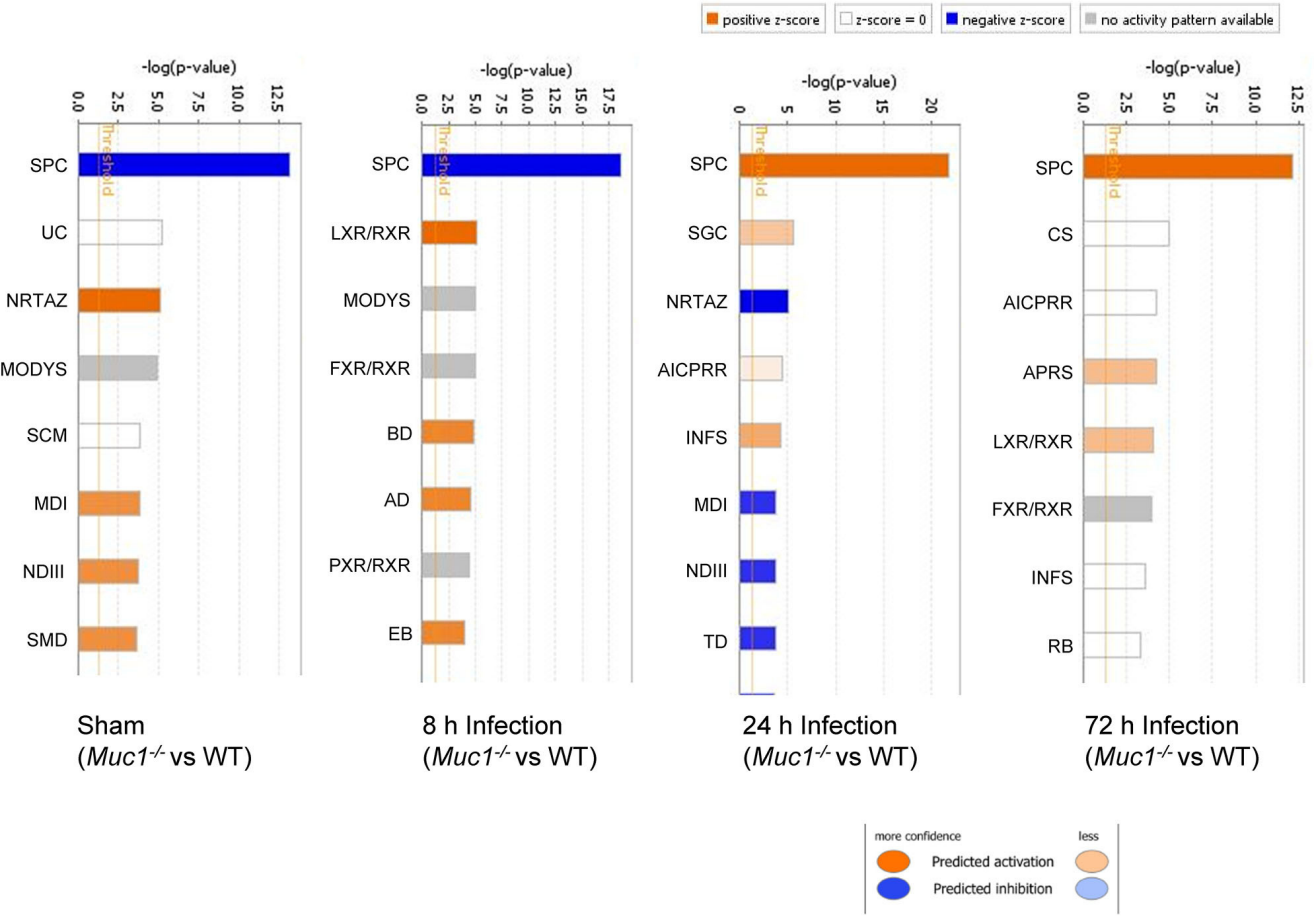
Ingenuity Pathway Analysis (IPA) showing the identified top canonical pathways of differentially expressed genes between *Muc1*^−/−^ vs. wild type (WT) gastric tissues in sham or in response to *H. pylori* infection over time. Significance *P*-values were calculated based on the Fisher's right tailed exact test. The-log (*p*-value) are shown on the top x-axis of the bar chart. The orange and blue colored bars indicate predicted pathway activation, or predicted inhibition, respectively (*z*-score). White bars are those with a *z*-score at or very close to 0. Gray bars indicate pathways, where no prediction can currently be made. IPA applies a-log (*p*-value) cutoff of 1.3 (threshold). SPC, SPINK1 Pancreatic Cancer Pathway; UC, Urea Cycle; NRTAZ, Neuroprotective Role of THOP1 in Alzheimer's Disease; MODYS, Maturity Onset Diabetes of Young Signaling; SCM, Superpathway of Citrulline Metabolism; MDI, Melatonin Degradation I; NDIII, Nicotine Degradation III; SMD, Superpathway of Melatonin Degradation; LXR/RXR, LXR/RXR Activation; FXR/RXR, FXR/RXR Activation; BD, Bupropion Degradation; AD, Acetone Degradation I (to Methylglyoxal); EB, Estrogen Biosynthesis; SGC, SPINK1 General Cancer Pathway; AICPRR, Activation of IRF by Cytosolic Pattern Recognition Receptors; INFS, Interferon Signaling; TD, Triacylglycerol Degradation; CS, Coagulation System; APRS, Acute Phase Response Signaling; RB, Retinol Biosynthesis.

In uninfected mice and at 8 h post-infection, there was significantly inhibition of the Serine Protease Inhibitor Kazal type 1 (SPINK1) pancreatic cancer pathway in the gastric tissue of *Muc1*^−/−^ mice compared with WT mice uninfected or at 8 h post-infection (*P* = 6.08E-14 and *P* = 2.25E-19, respectively, Figure 5), but at 24 and 72 h post-infection, we detected a strong activation of this pathway in *Muc1*^−/−^ mice (*P* = 1.38E-22 and *P* = 5.87E-13, respectively, Figure 5). The main physiological role of SPINK1 is to serve as a first line inhibitor of trypsin in the event of premature trypsinogen activation (Supplementary Figure 1). In addition, severe infections and tissue destruction cause elevation of SPINK1 in serum and urine, suggesting that it is an acute phase protein of the immune system. SPINK1 is highly expressed by the mucus-producing cells of the normal gastric mucosa and has been hypothesized to suppress proteolytic digestion of secreted mucus and promote gastric healing after injury (Marchbank et al., 1996, 1998; Konturek et al., 1998). In mouse, the homologous gene is designated *Spink3* (Wang and Xu, 2010).

The significant change in expression of genes related to the SPINK1 pathway included genes encoding proteins involved with digestion, absorption and secretion. Examples include chymotrypsin like elastase family (Cea1, Cela3b), carboxypeptidase (members Cpa1, Cpa2, Cpb1), chymotrypsin (members Ctrb2, Ctrc, Ctrl), Kallikrein related peptidase 3 (Klk3), and serine protease (Prss2, Prss3) (Table 4 and Supplementary Figure 2). In WT mice, the focal genes in the SPINK1 pathway were up-regulated most at 24 h post-infection and appeared to be switched off and were no longer differentially expressed at 72 h infection (Table 4), whereas in Muc1^−/−^ mice, the focal genes were up-regulated as early as 8 h after infection and their expression levels switched to 4–8-fold decreased expression toward 24 h and continuing to be further down-regulated at 72 h post-infection (most of the genes were decreased in expression by 8–32-fold, Table 4). In addition, there was higher expression of Spink3 in Muc1^−/−^ mice compared with WT mice at sham and 8 h infection, but Spink3 was downregulated at 24 and 72 h post-infection (Supplementary Figure 4).

**Table 4:** SPINK1 pancreatic cancer pathway.

		**Expr log ratio**											
**Symbol**	**Entrez gene name**	**Sham (KO/WT)**	**8 h (KO/WT)**	**24 h (KO/WT)**	**72 h (KO/WT)**	**WT (8 h/0)**	**WT (24 h/0)**	**WT (72 h/0)**	**KO (8 h/0)**	**KO (24 h/0)**	**KO (72 h/0)**	**Location**	**Type(s)**
1810009J06Rik/Gm2663	Trypsinogen 4	−0.056	1.466	0.098	−0.2	−0.32	0.155	−0.25	1.201	0.309	−0.4	Other	Other
2210010C04Rik	Trypsinogen 7	1.471	3.431	−3.51	−3.354	−0.83	1.839	−0.23	1.127	−3.14	−5.05	Extracellular space	Other
CELA1	Chymotrypsin like elastase 1	0.836	2.245	−1.95	−0.808	−0.37	1.141	−0.23	1.04	−1.65	−1.87	Extracellular space	Peptidase
CELA3B	Chymotrypsin like elastase 3B	1.603	3.467	−3.466	−1.881	−0.46	2.183	0.058	1.403	−2.89	−3.43	Other	Peptidase
CPA1	Carboxypeptidase A1	1.363	3.742	−3.94	−3.481	−1.15	1.743	−0.32	1.233	−3.56	−5.16	Extracellular space	Peptidase
CPA2	Carboxypeptidase A2	1.888	3.815	−3.898	−2.796	−0.79	2.522	0.277	1.133	−3.26	−4.41	Extracellular space	Peptidase
CPB1	Carboxypeptidase B1	1.639	3.857	−3.856	−3.764	−1.21	2.028	−0.05	1.008	−3.47	−5.45	Extracellular space	Peptidase
CTRB2	Chymotrypsinogen B2	1.532	3.417	−3.444	−4.267	−0.62	2.177	0.353	1.269	−2.8	−5.45	Cytoplasm	Peptidase
CTRC	Chymotrypsin C	1.088	2.284	−2.321	−0.605	−0.47	1.236	−0.14	0.723	−2.17	−1.83	Extracellular space	Peptidase
CTRL	Chymotrypsin like	1.575	3.656	−3.485	−3.223	−0.72	2.125	0.283	1.364	−2.94	−4.51	Extracellular space	Peptidase
KLK3	Kallikrein related peptidase 3	1.102	1.304	−1.047	0.584	0.468	1.084	−0.86	−0.58	−1.07	−1.18	Extracellular space	Peptidase
Prss1 (includes others)	Protease, serine 1 (trypsin 1)	−0.283	1.296	−1.273	0.212	−0.09	0.986	−0.39	1.669	0.152	0.11	Extracellular space	Peptidase
PRSS2	Serine protease 2	1.486	3.177	−2.882	−1.491	−0.49	2.044	−0.03	1.197	−2.32	−3.01	Extracellular space	Peptidase
PRSS3	Serine protease 3	1.397	3.224	−2.96	−1.316	−0.32	1.654	−0.29	1.503	−2.7	−3.01	Extracellular space	Peptidase

*Greater than 2 fold changes are highlight; –number in green with green filled indicates decreased expression and otherwise/red indicates increased expression. Number in green indicates decreased expression and red indicates increased expression*.

### Lipid Metabolism and Transport of Molecules

**Figure 6:**
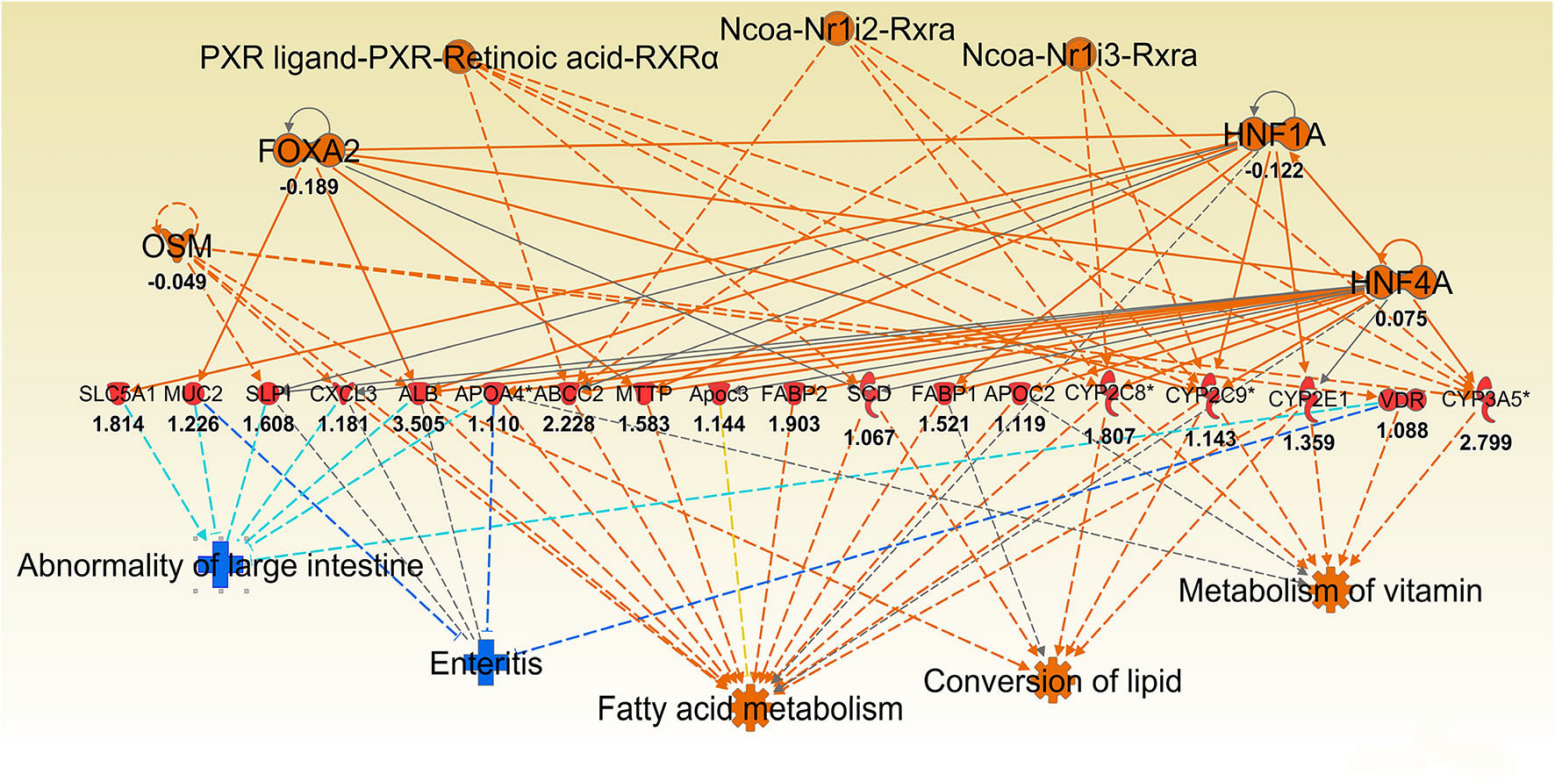
Ingenuity Pathway Analysis (IPA) top regulator effect networks analysis of differentially expressed (DE) genes between Muc1^−/−^ vs. wild type gastric tissues at 8 h H. pylori infection. Log ratio fold changes of DE genes is shown underneath each gene (red color symbol indicates increased expression). Prediction legend is the same as for Figure 3.

Consistent with observation of MUC1-suppressed lipid metabolism in uninfected mice, 8 h infection further induced expression of a wide range of lipid transporters in the gastric tissue of Muc1^−/−^ mice compared with WT (Figure 6). The IPA regulator effect analysis revealed that the categories most impacted by MUC1 deficiency were fatty acid metabolism, lipid conversion and metabolism of vitamin with consistence score = 16,028, followed by transport molecule (Table 2). In addition to increased expression of the genes leading to activated fatty acid metabolism that we observed in the uninfected mucosa (***Alb***, ***Abcc2***, Mttp, Apoc3, Fabp2, Fabp1), we also observed the increased expression of genes involved in the conversion of lipid and metabolism of vitamins (Cxcl3, Apoa2, Scd, and Vdr) and cytochrome P450 members (Cyp2c8, Cyp2c9, Cyp2e1) (Figure 6). For example, stearoyl-coA desaturase (Scd) is an endoplasmic reticulum (ER) enzyme that catalyzes the biosynthesis of monounsaturated fatty acids from saturated fatty acids. Cytochrome P450 encompasses a family of enzymes which oxidize steroids, fatty acids, and xenobiotics, and are important for the clearance of various compounds, as well as for hormone synthesis and breakdown. The expression of these genes is regulated by nuclear hormone receptors of the pregnane X receptor (PXR) family (Figure 6).

**Figure 7:**
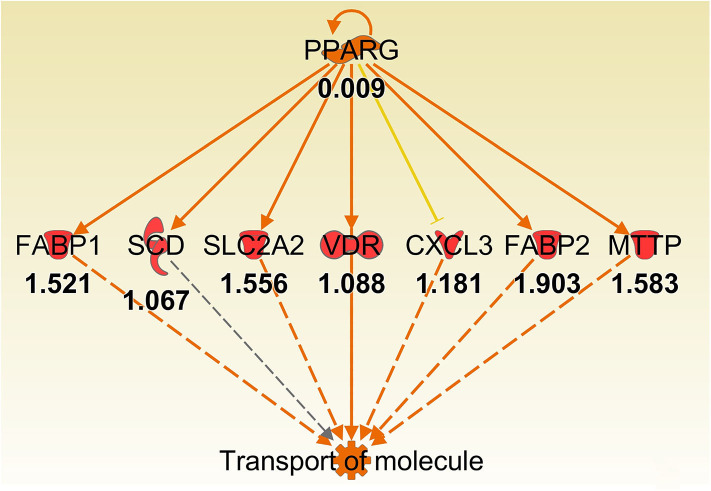
Ingenuity Pathway Analysis regulator effect networks analysis of differentially expressed (DE) genes between Muc1^−/−^ vs. wild type gastric tissues at 8 h H. pylori infection showed PPARG up-regulated transport of molecule pathway. Log ratio fold changes of DE genes is shown underneath each gene (red color symbol indicates increased expression). Prediction legend is the same as for Figure 3.

Some of the up-regulated genes in the fatty acid metabolism function category were also assigned to the transport molecule category, such as *Fabp1, Scd*, and *Cxcl3* plus additional genes *Slc2a2, Vdr, Fabp2* and microsomal triglyceride transfer protein gene (*Mttp*) (Figure 7). IPA indicated that expression of this set of genes is likely to be controlled by PPARG at 8 h post-infection (Figure 7). In addition, higher expression of solute carrier family genes (*Slc13a1, Slc26a3, Slc2a2, Slc40a1, Slc5a1*, and *Slc6a19*) was seen in the gastric tissue of *Muc1*^−/−^ mice compared with WT at sham and 8 h infection (Table 5). This family of genes plays a pivotal role in transport of a wide variety of solutes, including glucose and amino acids. However, *H. pylori* infection resulted in ~2–6-fold induction of most of these genes in WT at 24 h, vs. a ~2-fold decreased expression in *Muc1*^−/−^ gastric tissues (Table 5), and a similar trend was seen at 72 h infection (Table 5).

**Table 5:** Transport molecules.

		**Expr log ratio**											
**Symbol**	**Entrez gene name**	**Sham (KO/WT)**	**8 h (KO/WT)**	**24 h (KO/WT)**	**72 h (KO/WT)**	**WT (8 h/0)**	**WT (24 h/0)**	**WT (72 h/0)**	**KO (8 h/0)**	**KO (24 h/0)**	**KO (72 h/0)**	**Location**	**Type(s)**
A1CF	APOBEC1 complementation factor	1.347	0.698	−1.079	0.132	0.394	1.555	1.253	−0.378	−0.871	−0.134	Nucleus	Other
ABCC2	ATP binding cassette subfamily C member 2	1.725	2.228	−1.332	−0.11	0.678	1.936	1.631	1.181	−1.122	−0.205	Plasma membrane	Transporter
ABCG5	ATP binding cassette subfamily G member 5	1.371	0.438	−0.871	0.167	0.708	1.321	1.128	−0.225	−0.921	−0.075	Plasma membrane	Transporter
ALB	Albumin	2.375	3.505	−0.39	3.673	0.071	−0.36	−0.396	1.201	−3.125	0.902	Extracellular space	Transporter
Apoc3	Apolipoprotein C–III	1.006	1.144	−0.592	−0.095	0.266	0.968	0.912	0.404	−0.63	−0.189	Extracellular space	Transporter
CDH17	Cadherin 17	2.465	1.963	−1.423	−0.191	0.639	2.572	2.432	0.137	−1.316	−0.223	Plasma membrane	Transporter
CLCA4	Chloride channel accessory 4	2.268	1.933	−1.586	−0.171	−0.464	2.362	2.144	0.187	−1.492	−0.442	Plasma membrane	Ion channel
Defa3 (includes others)	Defensin, alpha, 3	2.169	1.852	−0.539	0.925	0.342	1.624	1.615	−0.189	−1.152	0.52	Extracellular space	Other
FABP1	Fatty acid binding protein 1	1.75	1.521	−1.483	0.244	0.102	1.613	1.52	−0.126	−1.619	0.015	Cytoplasm	Transporter
FABP2	Fatty acid binding protein 2	1.492	1.903	−1.491	−0.29	0.032	1.513	1.557	0.444	−1.469	−0.225	Cytoplasm	Transporter
GAL	Galanin and GMAP prepropeptide	1.15	1.07	−0.396	−0.221	−0.265	0.879	0.218	−0.346	−0.668	−1.153	Extracellular space	Other
GRP	Gastrin releasing peptide	1.312	0.395	−0.562	−0.013	0.216	1.143	0.769	−0.7	−0.731	−0.556	Extracellular space	Growth factor
GUCA2B	Guanylate cyclase activator 2B	1.281	1.412	−0.757	−0.231	−0.402	1.132	0.976	−0.272	−0.907	−0.537	Extracellular space	Other
MTTP	Microsomal triglyceride transfer protein	2.007	1.583	−1.268	−0.242	0.597	2.024	2.076	0.173	−1.25	−0.173	Cytoplasm	Transporter
PIGR	Polymeric immunoglobulin receptor	1.184	1.073	−0.166	0.283	0.138	0.852	1.143	0.027	−0.498	0.242	Plasma membrane	Transporter
SLC13A1	Solute carrier family 13 member 1	2.658	3	−1.167	−0.373	0.124	2.391	2.478	0.466	−1.434	−0.553	Plasma membrane	Transporter
SLC22A25	Solute carrier family 22 member 25	1.192	0.586	0.501	0.575	0.473	0.198	0.401	−0.353	−0.656	−0.501	Other	Other
SLC26A3	Solute carrier family 26 member 3	2.554	1.924	−1.685	−0.189	0.473	2.415	2.315	−0.157	−1.823	−0.428	Plasma membrane	Transporter
SLC2A2	Solute carrier family 2 member 2	1.008	1.556	−0.645	−0.138	0.328	1.118	1.199	0.876	−0.535	0.053	Plasma membrane	Transporter
SLC40A1	Solute carrier family 40 member 1	1.099	1.363	−1.16	−0.335	0.176	1.39	1.11	0.441	−0.868	−0.324	Plasma membrane	Transporter
SLC5A1	Solute carrier family 5 member 1	1.212	1.814	−0.525	−0.029	0.432	0.995	1.107	1.035	−0.742	−0.134	Plasma membrane	Transporter
SLC6A19	Solute carrier family 6 member 19	1.151	1.207	−1.227	−0.405	0.376	1.321	1.11	0.432	−1.058	−0.446	Plasma membrane	Transporter
TAC1	Tachykinin precursor 1	1.132	−0.012	−0.687	0.059	0.417	1.136	0.681	−0.727	−0.683	−0.392	Extracellular space	Other
ZG16	Zymogen granule protein 16	1.697	2.714	−2.265	−0.733	−0.141	2.164	1.229	0.876	−1.798	−1.202	Extracellular space	Other

*Greater than 2 fold changes are highlight; –number in green with green filled indicates decreased expression and otherwise/red indicates increased expression. Number in green indicates decreased expression and red indicates increased expression*.

### Antimicrobial Response and Inflammatory Response

One of the hallmarks of *H. pylori* colonization is the induction of a strong local antimicrobial and proinflammatory response by the infected epithelium. This initiates the mucosal infiltration of monocytes, neutrophils, and lymphocytes and the development of gastritis (McGuckin et al., 2007). Analysis of the DE genes between *Muc1*^−/−^ vs. WT in response to the infection revealed significant enrichment of functional clusters that were associated with antimicrobial and inflammatory responses.

*H. pylori* infection for 8 h resulted in ~4-fold induction of the regenerating islet-derived (REG) gene family 1B (*Reg1b*) mRNA in *Muc1*^−/−^ vs. no change in WT gastric tissues (Table 1). REG1B protein is highly expressed in several human pathologies, such as inflammatory bowel disease, many of which are associated with epithelial inflammation (van Beelen Granlund et al., 2013), indicating that the gastric mucosa of *Muc1*^−/−^ mice is more inflamed than WT mice within 8 h of infection. *H. pylori* infection for 24 h resulted in ~2.5-fold induction of mRNA for *Slfn12*, a schlafen family member, in *Muc1*^−/−^ vs. no change in WT gastric tissues. SLFN12 functions as an inducer of immune responses and is implicated in enterocyte differentiation (Table 1).

**Figure 8:**
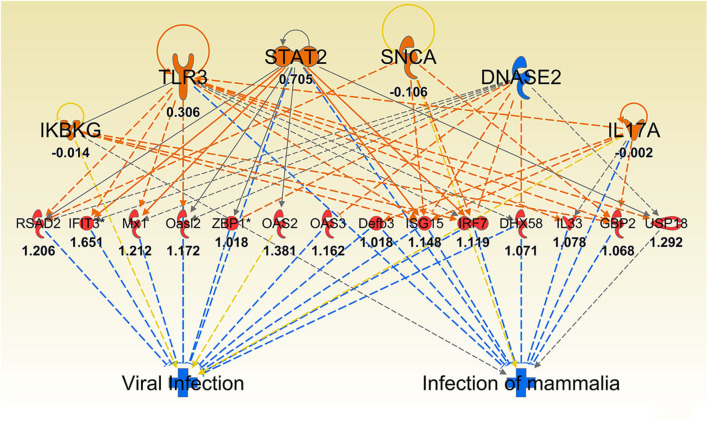
Ingenuity Pathway Analysis regulator effect networks analysis of differentially expressed (DE) genes between *Muc1*^−/−^ vs. wild type gastric tissues at 72 h *H*. pylori infection. Log ratio fold changes of DE genes in our data set is shown underneath each gene (red color symbol indicates increased expression). Prediction legend is the same as for Figure 3.

As infection progressed (24 and 72 h), we observed interferon gamma (IFNG)- regulated pathways were more strongly activated in *Muc1*^−/−^ vs. WT gastric tissues (*Z*-score 4.12, *P* = 1.65-E7, and *Z*-score 4.96, *P* = 5.7-E15, respectively, highlighted in bold in Table 6) characterized by the higher expression of many proinflammatory genes. Altered genes included: *Cmpk2, Defb3, Duoxa2, Fgg, Gbp2, Gbp3, Gbp4, Hspa1a/Hspa1b, Ido1, Ifi202b, Ifi44, Ifit1b, Ifit3, Igtp, Irf7, Irgm1, Isg15, Mx1, Mx2, Nlrc5, Oas2, Oas3, Rsad2, Rtp4, Serpina1, Slfn12l, Tap1, and Usp18* (Supplementary Table 3). Some of these genes are controlled by *Dnase2, Ikbkg, Il17a, Snca, Stat2, Tlr3* and their induction is associated with suppression of infection (Table 2 and Figure 8).

**Table 6:** Gastric *Muc1*^−/−^ vs. WT (KO/WT) upstream regulators^*^ at different time points.

**Time**	**Upstream regulator**	**Expr log ratio**	**Molecule type**	**Predicted activation state**	**Activation *z*-score**	***p*-value of overlap**
Sham (KO/WT)	HNF4A	0.235	Transcription regulator	Activated	3.443	0.00228
8 h (KO/WT)	HNF4A	0.075	Transcription regulator	Activated	3.477	0.0000242
8 h (KO/WT)	HNF1A	−0.122	Transcription regulator	Activated	3.791	2.62E-10
24 (KO/WT)	IRF7	1.178	Transcription regulator	Activated	4.874	6.48E-29
24 (KO/WT)	Interferon alpha		Group	Activated	4.386	6.02E-14
**24 (KO/WT)**	**IFNG**	**0.105**	**Cytokine**	**Activated**	**4.121**	**1.65E-07**
24 (KO/WT)	IRF3	−0.089	Transcription regulator	Activated	4.007	6.07E-24
24 (KO/WT)	TNF	−0.625	Cytokine	Activated	3.9	0.00368
24 (KO/WT)	IFN Beta		Group	Activated	3.81	8.62E-16
24 (KO/WT)	STAT1	1.008	Transcription regulator	Activated	3.982	9.03E-21
**72 (KO/WT)**	**IFNG**	**−0.328**	**Cytokine**	**Activated**	**4.956**	**5.7E-15**
72 (KO/WT)	Interferon alpha		Group	Activated	4.269	9.96E-18
72 (KO/WT)	IFN Beta		Group	Activated	3.54	3.91E-16
72 (KO/WT)	Ifn		Group	Activated	3.384	4.15E-17
72 (KO/WT)	Ifnar		Group	Activated	3.937	1.37E-22
72 (KO/WT)	STAT1	0.834	Transcription regulator	Activated	4.233	1.83E-24
72 (KO/WT)	IL21	0.094	Cytokine	Activated	3.606	3.41E-14
72 (KO/WT)	APP	0.143	Other	Activated	3.484	2.7E-07
72 (KO/WT)	IRF7	1.119	Transcription regulator	Activated	4.794	1.32E-33
72 (KO/WT)	TLR9	0.095	Transmembrane receptor	Activated	3.341	1.24E-12
72 (KO/WT)	IRF3	0.291	Transcription regulator	Activated	4.383	1.07E-24
72 (KO/WT)	IFNB1	0.126	Cytokine	Activated	3.65	7.79E-21
72 (KO/WT)	IFNA2		Cytokine	Activated	3.772	1.11E-15
72 (KO/WT)	TLR3	0.306	Transmembrane receptor	Activated	3.526	2.21E-16
24 (KO/WT)	ACKR2	0.246	G-protein coupled receptor	Inhibited	−3.606	9.71E-19
24 (KO/WT)	IL4	−0.108	Cytokine	Inhibited	−3.861	6.65E-08
24 (KO/WT)	SOCS1	0.136	Other	Inhibited	−3.41	1.73E-12
24 (KO/WT)	PTGER4	0.021	G-protein coupled receptor	Inhibited	−3.729	1.41E-12
24 (KO/WT)	TRIM24	−0.025	Transcription regulator	Inhibited	−3.627	2.93E-19
24 (KO/WT)	SIRT1	−0.335	Transcription regulator	Inhibited	−3.838	6.84E-19
24 (KO/WT)	PNPT1	0.491	Enzyme	Inhibited	−4.123	5.54E-25
72 (KO/WT)	ACKR2	0.188	G-protein coupled receptor	Inhibited	−3.317	8.73E-18
72 (KO/WT)	PTGER4	−0.141	G-protein coupled receptor	Inhibited	−3.44	5.67E-13
72 (KO/WT)	TRIM24	−0.032	Transcription regulator	Inhibited	−3.978	1.06E-21
72 (KO/WT)	SIRT1	0.232	Transcription regulator	Inhibited	−4.243	2.51E-17
72 (KO/WT)	PNPT1	0.315	Enzyme	Inhibited	−3.873	6.15E-25

Upstream Regulators^*^: Use IPA Upstream Regulator Analysis to identify the upstream regulators that may be responsible for gene expression changes observed in your experimental dataset. IPA predicts which upstream regulators are activated or inhibited to explain the up-regulated and down-regulated genes observed in your dataset. Knowledge of this regulatory cascade can help you understand the biological activities occurring in the tissues or cells that you are studying. IPA uses a z-score algorithm to make predictions. The z-score algorithm is designed to reduce the chance that random data will generate significant predictions.

### Verification of IPA Identified Canonical Pathways and Networks

To further compare the wild type and *Muc1*^−/−^ response to infection, we also performed a sample-to-sample analysis using Graphia Pro network analysis software, with the filtered set of 6016 probesets showing at least 1.5-fold difference between highest and lowest across the eight samples (representing 4,448 unique genes as well as 1,305 unannotated probesets). A Pearson correlation coefficient threshold of 0.95 was used as it was the highest value to include all eight samples in the network. Supplementary Figure 3 shows that the *Muc1*^−/−^ 24 and 72 h samples were separated from all other samples on the basis of gene expression patterns, and that the earlier *Muc1*^−/−^ samples were more similar to the wild type 24 and 72 h samples that than earlier wild type samples. This suggested that there were distinct gene expression patterns differentiating the response of the *Muc1*^−/−^ animals from that of the wild type animals, consistent with the DE gene analysis. We therefore constructed a GCN using a Pearson correlation coefficient threshold of 0.87 which included all 6016 probesets of the filtered list (making 78,064 edges) in the analysis. A summary of the expression patterns of the largest clusters is shown in Supplementary Table 4 and a full list of clusters and histograms showing the average expression pattern of clusters with 10 or more nodes is available in Supplementary Table 5.

We searched the GCN for known proliferation markers, mitochondrial genes and protein synthesis genes and found no indication that these functions were disrupted in the *Muc1*^−/−^ response to the infection.

The DE genes described above were concentrated in three clusters of the GCN. The genes which were upregulated in the *Muc1*^−/−^ sham infected animals compared to the wild type sham infected and also in the wild type at 72 h post infection compared to the wild type sham infected were predominantly in Cluster004 (Supplementary Figure 4). Genes in this cluster showed enrichment of GO terms relating to digestion, transport, intestinal absorption, microvillus, brush border membrane. DE genes which were upregulated in 24 and 72 h *Muc1*^−/−^ samples compared with the corresponding wild type samples, and some of the genes that were higher in *Muc1*^−/−^ at 72 h compared with *Muc1*^−/−^ sham infected, were mainly in Cluster005 (Supplementary Figure 4), enriched for GO terms associated with innate immune response and response to pathogen. The set of DE genes which were higher in *Muc1*^−/−^ 8 h than wild type 8 h, but lower in *Muc1*^−/−^ 24 and 72 h compared to the corresponding wild type time points and also lower in *Muc1*^−/−^ 72 h compared to *Muc1*^−/−^ sham were in Cluster008 (Supplementary Figure 4). This showed enrichment for GO terms related to catabolism, digestion and extracellular space. This cluster also included *Spink3*, identified as a key regulator by the IPA analysis (Supplementary Figure 4), and genes encoding many members of the SPINK1 pathway, including *Cela3b, Cpa1, Cpa2, Cpb1, Ctrc, Ctrl, Prss2*, and *Prss3* (Supplementary Figure 1). This cluster also contained a number of other proteases and protease inhibitors.

Analysis of the expression pattern of Muc1 showed that the Muc1^−/−^ samples had very low levels of Muc1 mRNA, as expected. Muc1 was found in Cluster020 where the average expression across all cluster genes of the Muc1^−/−^ samples was half that of the wild type and there was no change during the infection in either WT or Muc1^−/−^ (Supplementary Figure 4). This cluster contains genes potentially directly affected by the lack of MUC1, and includes the Srp54b and Srp54c genes, encoding signal recognition particles involved in the export of proteins and both reduced to about 50% of their wild type expression, Rfx6 (regulatory factor X, 6), and sodium channel genes Scn2a and Scn7a. This cluster was enriched for the GO MF term receptor regulatory activity and the GO CC terms vesicle and extracellular region.

In the GCN analysis, Cluster007 showed reduced expression overall in Muc1^−/−^ compared to wild type sham and also reduced further as the infection progressed (Supplementary Figure 4). This cluster contained Acta1, encoding smooth muscle actin, as well as the actin crosslinking protein gene Actn3 and two myosin genes (Myo1h and Myh2), suggesting that both infection and Muc1 knockout disrupt the cytoskeleton. The role of H. pylori in actin rearrangement has been described (Tohidpour et al., 2017) and this observation warrants further investigation.

### Selected DE Genes From Diverse Gene Ontologies Showed Consistent Trends in RT-qPCR Assay

**Figure 9:**
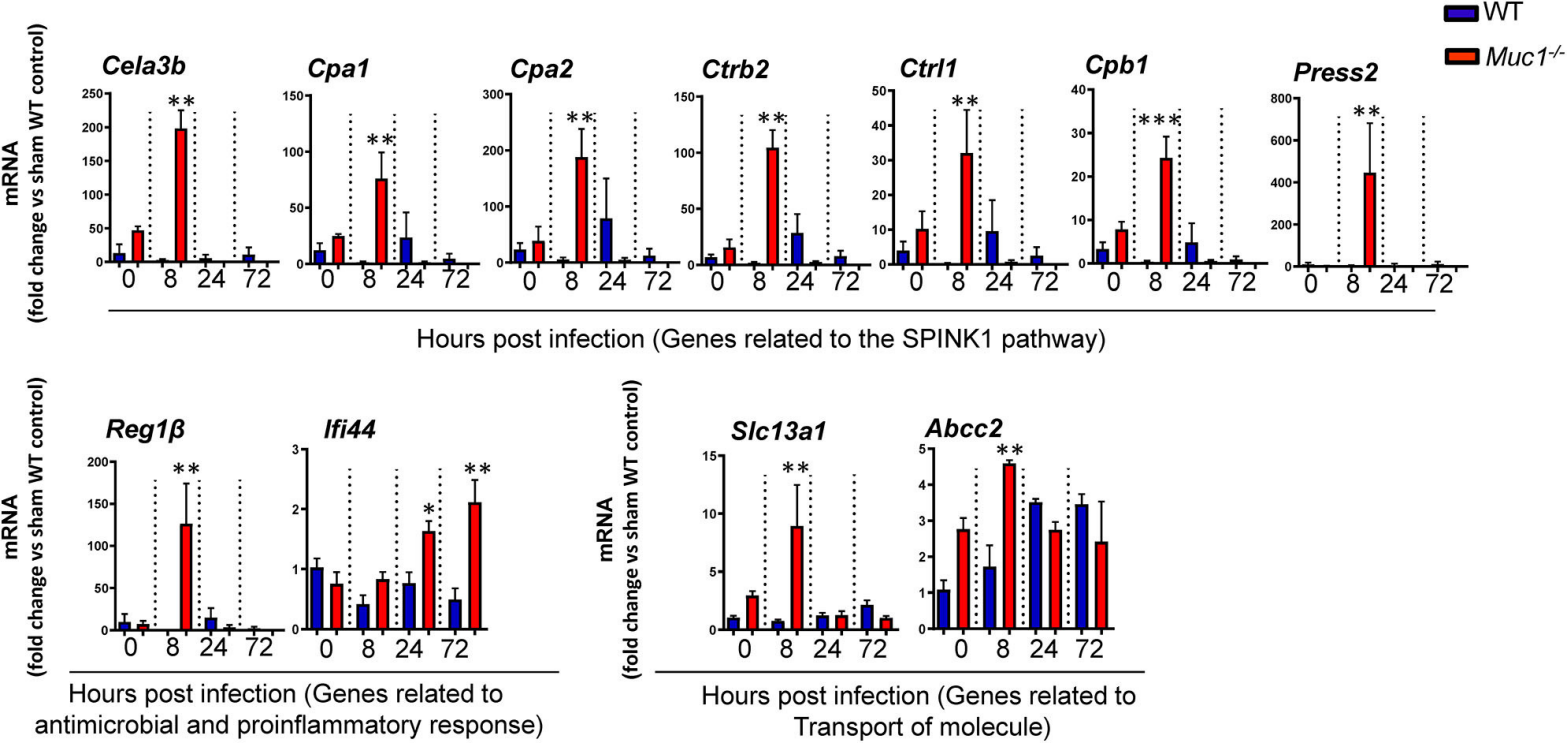
RT qPCR validation of transcripts selected as the most highly differentially expressed genes between *Muc1*^−/−^ vs. wild type (WT) gastric tissues. Statistics: Ordinary one-way ANOVA corrected for multiple comparisons by controlling the False Discovery Rate (FDR) using “Two-stage step-up method of Benjamini, Krieger, and Yekutieli;” *n* = 3, * vs. WT at each time point, *P < 0.05, **P < 0.01, ***P < 0.001).

Several genes identified as highly DE at one or more time-points over 72 h based on microarray analysis and which were also part of significantly altered pathways and networks identified by IPA were independently validated by RT-qPCR (Figure 9). Overall, most genes measured by qPCR confirmed the changes in gene expression identified by microarray (Figure 9).

## Discussion

In this study we first identified a specific set of MUC1-regulated gastric genes by performing microarray analysis of the gastric transcriptome of *Muc1* knockout mice under physiological conditions. Gene categorization analysis (using both IPA and GO term enrichment) indicated that in the uninfected state MUC1-associated genes are involved in a wide range of cellular functions, with the most highly impacted network related to lipid transport and regulated via the transcription factors HINF1A, HINF4A, and SOX2 (Figures 3, 4). The Sry-containing protein SOX2 was initially shown to regulate the self-renewal of mouse and human embryonic stem cells (ESCs) and controls the formation of several cell types during fetal development, such as anterior foregut endoderm (Que et al., 2007). SOX2 is also important for the maintenance of stem cells in multiple adult tissues, and it is one of the key transcription factors for establishing induced pluripotent stem cells (Boyer et al., 2005). SOX2 has been shown to bind the *MUC1* promoter in human embryonic stem cells (Boyer et al., 2005). In cancer cells, MUC1 has been shown to drive self-renewal capacity and promote stemness of cancer cells. Targeting the MUC1 cytoplasmic domain (MUC1-C) genetically or pharmacologically decreased: (i) expression of breast cancer stem cell markers including SOX2 (Hata et al., 2019); and (ii) lung cancer stem cell generation associated with decreased levels of SOX2 (Ham et al., 2016). Our study further demonstrates that MUC1 deficiency decreases the expression of *Sox2* in normal gastric tissue. Further studies will now be needed to define how Muc1 influences *Sox2* expression.

Knowledge of the gastric microbiota is evolving, and it is now appreciated that the stomach supports a bacterial community with possibly hundreds of bacterial species that influence stomach homeostasis (Sheh and Fox, 2013). Most of these microbial residents will grow within the mucus layer that overlies the gut epithelium. The mucus layer in the stomach consists of a cell-associated layer (predominantly MUC1) and secreted mucins (mainly MUC5AC) (McGuckin et al., 2011). Recent studies showed that the maturation and function of the mucus layer are strongly influenced by the gut microbiota. In turn, the glycan repertoire of mucins can select for distinct mucosa-associated bacteria that are able to bind or degrade specific mucin glycans as a nutrient source (Schroeder, 2019). It is likely that gastric microbial ecosystems will differ between *Muc1*^−/−^ and WT mice. Alterations in the mucosa-associated microbiota could impact on host nutrient metabolism, thus explaining the higher expression of lipid transporter genes in *Muc1*^−/−^ gastric tissue. MUC1 has also previously been shown to be a metabolic master regulator in cancer cells in which it regulated metabolism flux at multiple levels, including: (i) directly regulating expression of metabolic genes by acting as co-transcriptional factor; (ii) regulating metabolic functions by modulating the activity/stability of enzymes and transcription factors; and (iii) modulating levels of reactive oxygen species and metabolite flux (Pitroda et al., 2009; Mehla and Singh, 2014). For example, MUC1-C has been shown to regulate glycolysis by directly modulating the functions of the metabolic enzyme, pyruvate kinase M2 (Kosugi et al., 2011). Our results suggest that MUC1 also modulates epithelial metabolic function in the normal gastric mucosa with potential importance in both health and during mucosal infection.

In addition to providing the first-line defensive barrier against many pathogens, gastric mucus also protects the gastric mucosa from enzymatic autodigestion (e.g., by pepsin) and from erosion by acid, premature trypsinogen activation, and from ingested caustic materials. An intriguing and novel finding of MUC1 deficiency under physiological conditions is the inhibition of the SPINK pancreatic cancer pathway. SPINK1 or its homologous protein SPINK3 in mouse, originally isolated from the pancreas as an inhibitor of trypsin, is present throughout the gastrointestinal tract (with stomach having the 2nd highest expression behind pancreas) (Marchbank et al., 1996), and it is the only protease inhibitor known to be secreted into the intestinal lumen. SPINK1 functions as an inhibitor of serine-proteinases, including trypsin, that prevents excessive digestion of the mucus by luminal proteases within the stomach and colon (Marchbank et al., 1996). The inhibition of this pathway in *Muc1*^−/−^ mice might result in a thinner mucus layer due to the high uninhibited serine proteinase activity, and thereby predispose to increased penetration by *H. pylori*. This mucus defect could explain the ~10-fold higher *H. pylori* colonization in *Muc1*^−/−^ mice compared with WT mice very early in infection at a time when innate responses to the bacteria would only be in the preliminary stages of activation and unlikely to impact on bacterial survival or replication. *H. pylori* secretes a serine protease, HtrA, that is involved in gastric colonization and pathogenesis (Zarzecka et al., 2019), and another possibility is that SPINK1 inhibits this protease and MUC1 deficiency-induced reduction of SPINK1 also enhances HtrA activity and pathogenesis. In addition, SPINK1 has been shown to accelerate the healing of stress-induced gastric lesions by inhibiting gastric acid and pepsin outputs in rats (Konturek et al., 1998). The greater bacterial burden in the MUC1-deficient mice continued at 24–72 h of infection, and we observe this pathway to be potently activated at 24 and 72 h of infection in the gastric tissue of *Muc1*^−/−^ mice compared with WT mice. The higher bacterial burden could cause more tissue damage in the gastric mucosa of *Muc1*^−/−^ mice compared with WT mice, thus the activation of this pathway is likely to stimulate repair at sites of infection and epithelial injury in *Muc1*^−/−^ mice. Induction of the pathway could also represent a feedback loop to prevent further damage of mucus due to the higher colonization of *H. pylori*. We previously have demonstrated that both epithelial and leucocyte MUC1 mucin protect against *H. pylori* pathogenesis in mice by limiting pathogen contact with the host epithelium and by limiting activation of the NLRP3 inflammasome in macrophages, respectively. We now show that MUC1 may also limit *H*. *pylori* penetration by protecting mucus from enzymatic autodigestion. Together these findings suggest that MUC1 protects the host against pathogens via multiple distinct mechanisms involving multiple cell types.

The time course analysis of this study showed the induction of a potent anti-pathogen response within 24 h of *H. pylori* infection, with infection in *Muc1*^−/−^ mice characterized by higher induction of INFG-regulated target genes (Supplementary Table 3). For instance, we observed that the genes encoding interferon induced protein 44 (*Ifi44* gene) and schlafen family member 12 like (*Slfn12l* gene) are two of the most highly induced genes in *Muc1*^−/−^ mice vs. WT at 24 h post-infection. IFI44, was associated with multiple different viral infections (Honda et al., 1990; Bochkov et al., 2010; Kaczkowski et al., 2012), and has been shown to induce an antiproliferative state in cells (Hallen et al., 2007). SLFN12L also functions as a regulator of cell proliferation and differentiation, and in the induction of immune responses (Mavrommatis et al., 2013). This finding suggested that there are more infected epithelial cells in the gastric mucosa of *Muc1*^−/−^ mice, and consequently a more robust response from the host to try to limit the infection by increased expression of immune response and antiproliferation genes and a higher IFNG-modulated anti-pathogen response. A higher number of infected cells is consistent with the higher *H. pylori* colonization in the gastric mucosa of *Muc1*^−/−^ mice throughout the first 3 days of infection. These data suggested that MUC1 has an anti-infection and anti-inflammatory function in response to *H. pylori* infection, which is consistent with our previous finding that MUC1 suppresses inflammation in response to *H. pylori* infection *in vitro* and *in vivo* (McGuckin et al., 2007; Linden et al., 2009; Sheng et al., 2013) via negative regulation of NF-κB (Guang et al., 2010; Sheng et al., 2013) and the NLRP3 inflammasome (Ng et al., 2016).

A sample-to-sample network of all samples in the analysis showed that the *Muc1*^−/−^ 24 and 72 h samples were separated from all others, indicating that they were substantially different from both the earlier *Muc1*^−/−^samples and the same time points in WT. In addition, sham and 8 h infected *Muc1*^−/−^ samples were more similar to the WT 24 and 72 h infection samples than the earlier time points in WT. This association was substantiated by the GCN analysis which revealed clusters of genes in which expression patterns were distinct in *Muc1*^−/−^ mice at 24 and 72 h infection, and *Muc1*^−/−^ sham and 8 h expression was similar to WT 24 and 72 h expression. These results suggest that *Muc1*^−/−^animals are poised to respond to the early stages of the infection, although by 72 h they are very different from the WT. These data highlight the importance of MUC1 for restriction of early *H. pylori* infection by alterations in the molecular network providing mucosal defense against infection.

In conclusion, we have reported a global overview of MUC1-regulated genes in response to *H. pylori* infection, it likely reflects the subsequent defensive response although this needs to be verified at the proteomic level.

## Data Availability Statement

The original contributions presented in the study are publicly available. This data can be found here: https://www.ncbi.nlm.nih.gov/geo/query/acc.cgi?acc=GSE151418.

## Ethics Statement

The animal study was reviewed and approved by The University of Melbourne Animal Ethics Committee approval (AEEC No. 06205).

## Author Contributions

YS designed, performed and analyzed mouse, RNA extraction, microarray and QPCR experiments, and wrote the manuscript. GN and AE designed, performed, and analyzed mouse experiments. GP designed, performed, and analyzed microarray experiments. KS performed the GCN analysis. SH provided intellectual input to the experimental design and analysis. PS provided intellectual input to the project including experimental design, detailed comments, and suggestions on drafts of the manuscript. MM supervised the project, provided intellectual input to experimental planning and analysis, was involved in writing, and was responsible for the final version of the manuscript. All authors contributed to the article and approved the submitted version.

## Conflict of Interest

The authors declare that the research was conducted in the absence of any commercial or financial relationships that could be construed as a potential conflict of interest.
