# Supplementary material for: Influence of the MUC1 Cell Surface Mucin on Gastric Mucosal Gene Expression Profiles in Response to Helicobacter pylori Infection in Mice
Source: Front Cell Infect Microbiol. 2020 Jul 24;10:343. doi: 10.3389/fcimb.2020.00343 (PMC7393270; doi:10.3389/fcimb.2020.00343)
Supplement: Supplementary file 2 [file Table_2.DOCX]

**Supplementary Table 2. Primer set for Q-PCR to detect the indicated mRNA levels**

|  | Forward primers | Reverse primers |
| --- | --- | --- |
| *Actb* | 5'-CTTCTTGGGTATGGAATCCTGTG-3' | 5'-AGCACTGTGTTGGCATAGAGGTC-3' |
| *Cela3b* | 5'- TGCATCTCGACTTCTCGCAC-3' | 5'- GGTGCACAAAGAGGTCTCCA-3' |
| *Cpa1* | 5’- CACCCACAAAACGAATCGCA-3’ | 5’- AGCATCCCAGTTCCTGTTGG-3’ |
| *Cpa2* | 5’- CCATGAGGTTGACCCCGTTA-3’ | 5’- CACCTCAAGAACTTGATCTCCCA-3’ |
| *Ctrb1* | 5’- GGGTCAAGACAACCGATGTG-3’ | 5’- TACGCACGGTGAAGGAGTTG -3’ |
| *Ctrl1* | 5’- ATCAGTGGTGTGGGCAATGT-3’ | 5’- CATGGCATCGGTAATGCGTG-3’ |
| *Cpb1* | 5'- TGTGAGAGAGGCTGTCCGTA -3' | 5'- CGTCAATGTTGACCACAGGC -3' |
| *Press2* | 5'- TCTGTGCTCATGACTTTCTGTCA -3' | 5'- TCCACAGGGAAAGCAACAGC -3' |
| *Reg1β* | 5’- GGGAGGCTGATCTCTTTTGC-3’ | 5’- AGGCCACAAAGTTGCTCTCA-3’ |
| *Ifi44* | 5’- TGGCATTCTGCATTTGGCTT-3’ | 5’- AATGCCTCCAGCTTGGACTT-3’ |
| *Slc13a1* | 5’- ACTTGGGCCAATGAGGTATCAA-3’ | 5’- ACCGACCAACCAGTGACAAA-3’ |
| *Abcc2* | 5’- TCTGCAACTCTACTTTTTGGAATCT-3’ | 5’- AAGCCCAAGGGAATCCACAC-3’ |
