# Supplementary material for: Influence of the MUC1 Cell Surface Mucin on Gastric Mucosal Gene Expression Profiles in Response to Helicobacter pylori Infection in Mice
Source: Front Cell Infect Microbiol. 2020 Jul 24;10:343. doi: 10.3389/fcimb.2020.00343 (PMC7393270; doi:10.3389/fcimb.2020.00343)
Supplement: Supplementary file 4 [file Table_4.DOCX]

| Cluster number | Number of nodes | Expression pattern |
| --- | --- | --- |
| 001 | 358 | KO 8hr > WT sham > all others |
| 002 | 238 | KO sham > all others |
| 003 | 226 | KO 24hr > all others |
| 004 | 198 | WT24hr, WT 72hr, KO sham, KO8 hr > KO 72 hr > all others |
| 005 | 159 | KO 24hr, KO 72hr > all others |
| 006 | 153 | KO 72hr > all others |
| 007 | 138 | WT sham > all others |
| 008 | 137 | KO 8hr > WT 24hr > KO sham > WT sham, WT 8hr, WT 72hr > KO 24hr, KO 72hr |
| 009 | 114 | WT sham > WT 8hr > WT 24hr, WT 72 hr > all KO samples |
| 010 | 104 | WT sham > all others |
| 011 | 102 | WT 24hr, KO sham > all others |
| 012 | 97 | WT sham, KO 24hr > WT 8hr, WT 72hr, KO 8hr, KO 72hr > all others |
| 013 | 74 | WT 72hr > all others |
| 014 | 70 | KO sham < all others |
| 015 | 66 | WT 8hr, KO sham < all others |
| 016 | 65 | KO 8hr, KO 24hr, KO 72hr < all others |
| 017 | 65 | WT 24hr > all others |
| 018 | 63 | Increases over time in WT; flat in KO |
| 019 | 62 | KO 8hr > all others |
| 020 | 62 | All WT > all KO |
| 021 | 59 | KO 72hr > WT 24hr, WT 72hr, KO sham, KO 8hr > all others |
| 022 | 58 | WT 72 hr > WT sham, WT 8hr, KO 8hr, KO 24hr, KO 72hr > WT 24hr > KO sham |
| 023 | 58 | KO 24hr > all others, KO 72hr < all others |
| 024 | 56 | KO 8hr, KO 24hr > all others, KO 72hr < all others |
| 025 | 56 | WT 8hr, KO 8hr > all others |
| 026 | 54 | WT 24hr < all others |
| 027 | 50 | KO 72hr > WT sham > all others |
| 028 | 50 | KO 8hr, KO 24hr > all WT, KO 72hr > KO sham |

**Supplementary Table 4:** Summary of GCN clusters. The expression pattern of clusters with at least 50 nodes is described.
