# Supplementary material for: Influence of the MUC1 Cell Surface Mucin on Gastric Mucosal Gene Expression Profiles in Response to Helicobacter pylori Infection in Mice
Source: Front Cell Infect Microbiol. 2020 Jul 24;10:343. doi: 10.3389/fcimb.2020.00343 (PMC7393270; doi:10.3389/fcimb.2020.00343)
Supplement: Supplementary file 6 [file Data_Sheet_1.PDF]

A

B

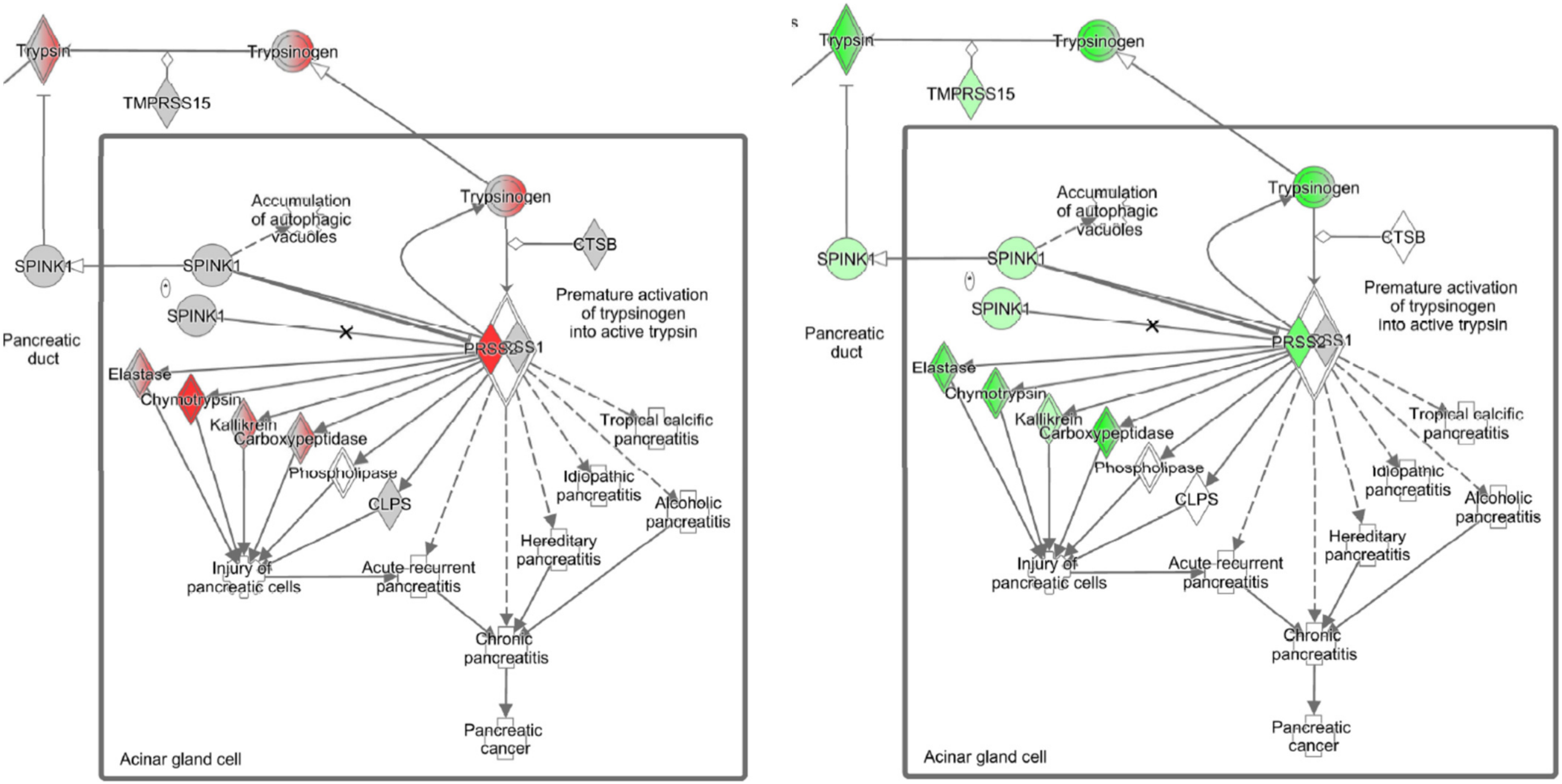


**Sup Figure 1:** Diagrams of SPINK 1 pancreatic cancer pathway between gastric *Muc1*$^{-/-}$ vs WT in response to *H. pylori* infection. (A) Increased the expression of the target genes are showed in red at 8 h infection. (B) Decreased the expression of the target genes are showed in green at 72 h infection.

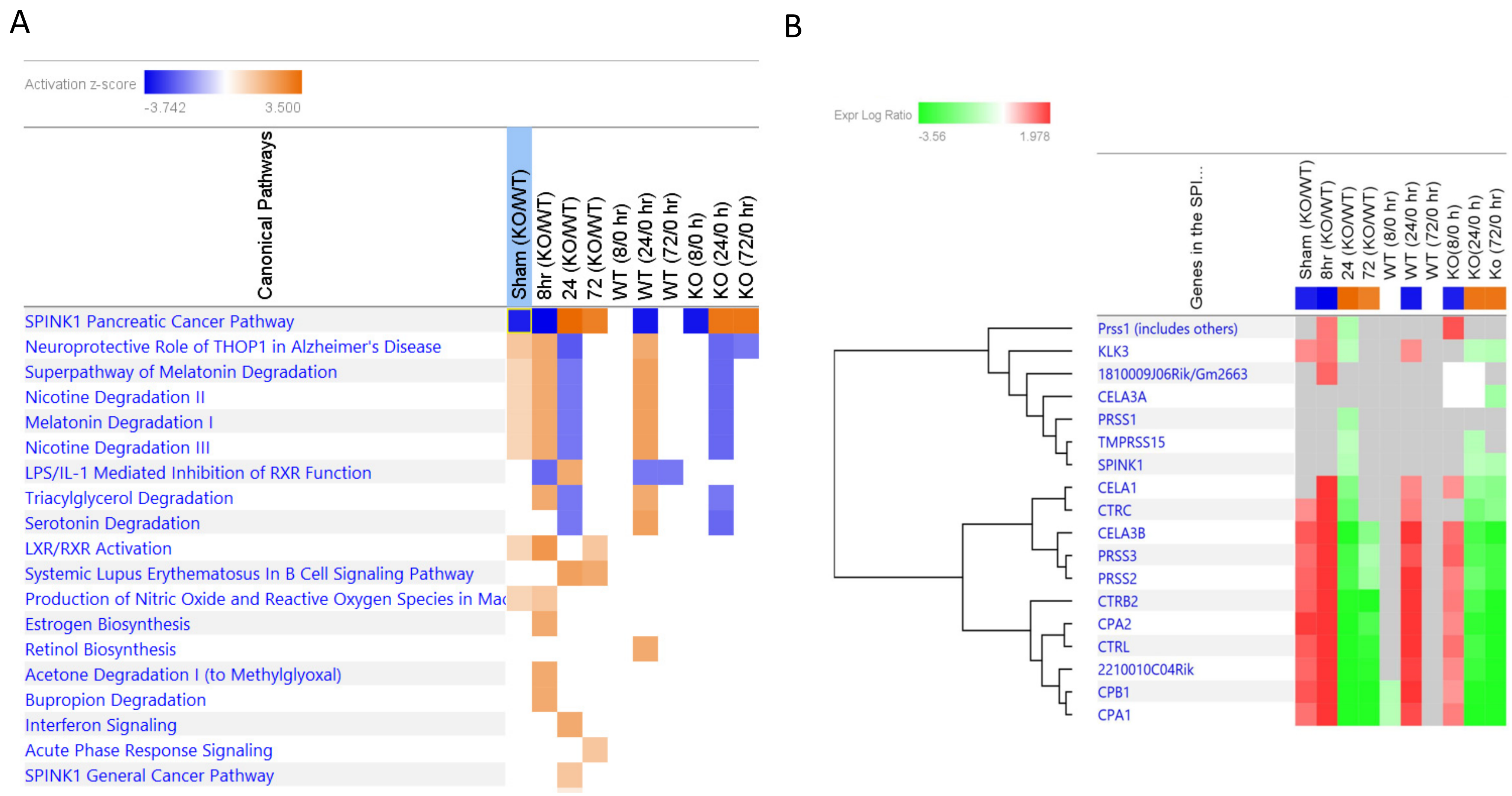


**Sup Figure 2:** (A) Z-scored heat map of canonical pathways associated with time-course of changes between gastric *Muc1−/−* vs WT (KO/ WT) or gastric WT mice or gastric *Muc1−/−(KO)* mice in response to *H. pylori* infection. (B) Heat map of DE genes associated with IPA terms canonical pathways associated with SPINK1 pancreatic cancer pathway in our data set.

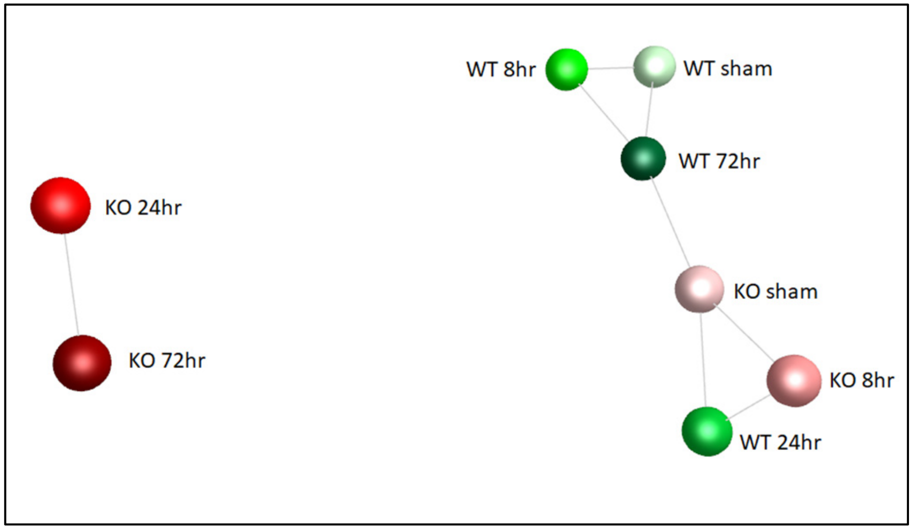


**Sup Figure 3:** Sample-to-sample analysis of the *H. pylori* infection time course in WT and *Muc1*$^{-/-}$ samples. In this analysis (similar to a principal components analysis) nodes (spheres) represent samples and the edges (lines) show correlations between samples of ≥ 0.95. The network layout shows the similarity of samples based on the expression of all genes in the sample.

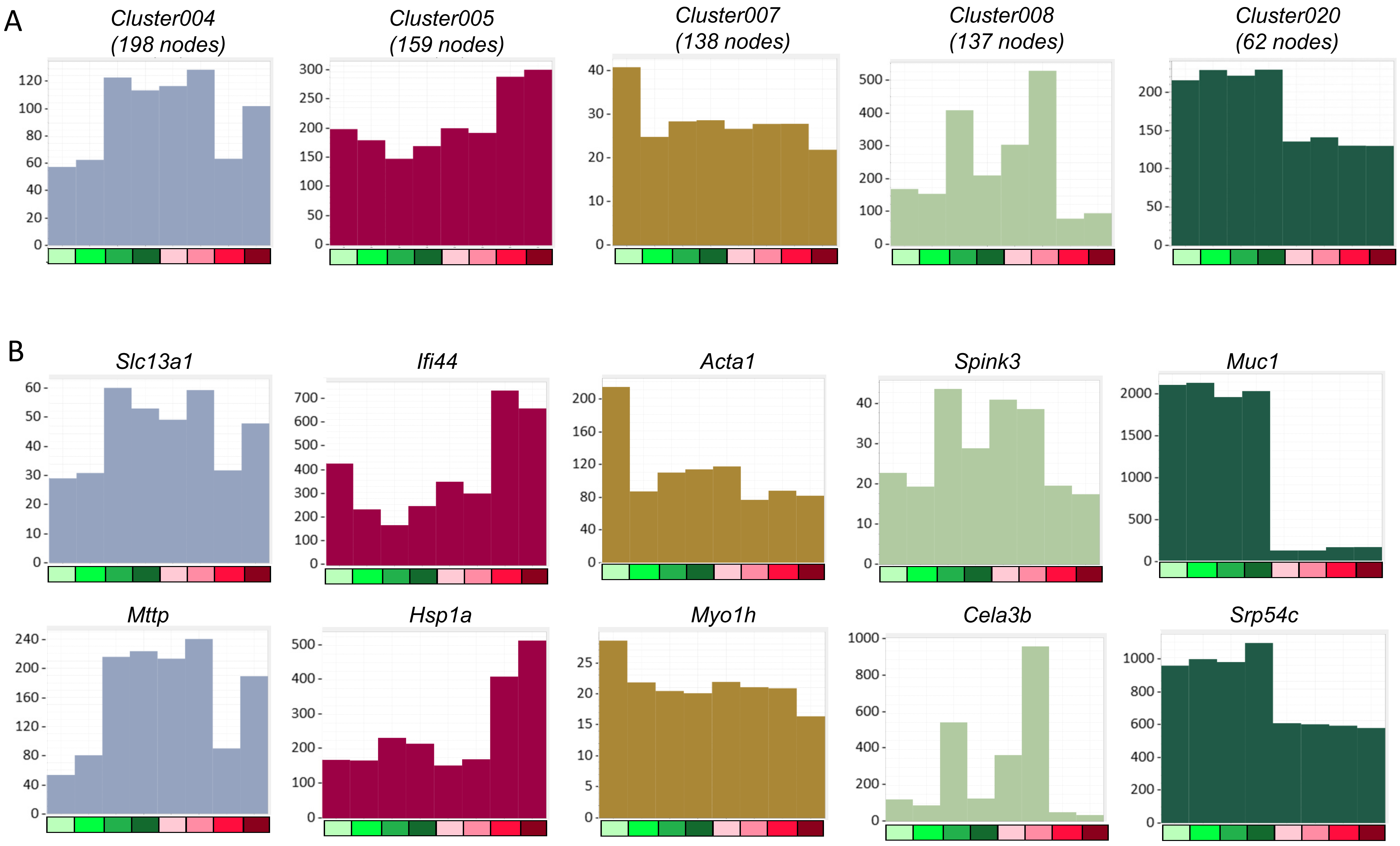


**Sup Figure 4:** Gene coexpression network analysis. The network was developed at a correlation coefficient threshold of 0.87 and clustering was performed using the Markov clustering algorithm at an inflation value of 1.7. X axis shows the time course samples colour coded as for Sup Figure 3; green – WT; red – *Muc1*$^{-/-}$. Y axis shows the expression (relative intensity). **(A)** The expression pattern of GCN cluster004, 005, 007, 008 and 020. **(B)** Examples of genes of interest from GCN cluster004, 005, 007, 008 and 020, coloured as for the clusters in **(A)**.
